# Construction and sequence sampling of deep-coverage, large-insert BAC libraries for three model lepidopteran species

**DOI:** 10.1186/1471-2164-10-283

**Published:** 2009-06-26

**Authors:** Chengcang Wu, Dina Proestou, Dorothy Carter, Erica Nicholson, Filippe Santos, Shaying Zhao, Hong-Bin Zhang, Marian R Goldsmith

**Affiliations:** 1 Department of Soil and Crop Sciences, Texas A&M University, College Station, TX 77843-2474, USA; 2 Department of Biological Sciences, University of Rhode Island, Kingston, RI 02881-0816, USA; 3 The Institute for Genomic Research, 9712 Medical Center Dr, Rockville, MD 20850, USA; 4 Current address: Lucigen Corporation, 2120 West Greenview Dr, Middleton, WI 53562, USA

## Abstract

**Background:**

*Manduca sexta, Heliothis virescens*, and *Heliconius erato *represent three widely-used insect model species for genomic and fundamental studies in Lepidoptera. Large-insert BAC libraries of these insects are critical resources for many molecular studies, including physical mapping and genome sequencing, but not available to date.

**Results:**

We report the construction and characterization of six large-insert BAC libraries for the three species and sampling sequence analysis of the genomes. The six BAC libraries were constructed with two restriction enzymes, two libraries for each species, and each has an average clone insert size ranging from 152–175 kb. We estimated that the genome coverage of each library ranged from 6–9 ×, with the two combined libraries of each species being equivalent to 13.0–16.3 × haploid genomes. The genome coverage, quality and utility of the libraries were further confirmed by library screening using 6~8 putative single-copy probes. To provide a first glimpse into these genomes, we sequenced and analyzed the BAC ends of ~200 clones randomly selected from the libraries of each species. The data revealed that the genomes are AT-rich, contain relatively small fractions of repeat elements with a majority belonging to the category of low complexity repeats, and are more abundant in retro-elements than DNA transposons. Among the species, the *H. erato *genome is somewhat more abundant in repeat elements and simple repeats than those of *M. sexta *and *H. virescens*. The BLAST analysis of the BAC end sequences suggested that the evolution of the three genomes is widely varied, with the genome of *H. virescens *being the most conserved as a typical lepidopteran, whereas both genomes of *H. erato *and *M. sexta *appear to have evolved significantly, resulting in a higher level of species- or evolutionary lineage-specific sequences.

**Conclusion:**

The high-quality and large-insert BAC libraries of the insects, together with the identified BACs containing genes of interest, provide valuable information, resources and tools for comprehensive understanding and studies of the insect genomes and for addressing many fundamental questions in Lepidoptera. The sample of the genomic sequences provides the first insight into the constitution and evolution of the insect genomes.

## Background

Large-insert bacterial artificial chromosome (BAC) libraries have been shown to be critical resources for many aspects of molecular and genomic studies [1,2], such as the positional cloning of genes [3] and quantitative trait loci [4], comparative studies of synteny and gene organization among different species [5], as well as for local or whole genome physical and genetic mapping and sequencing [6-11]. Arrayed, large-insert DNA libraries have provided the opportunity for researchers to analyze and share information and resources on specific clones [1,2,12,13]. Hundreds of BAC libraries have been constructed for microbe, plant and animal species [1,2,6,7,12,13]. However, only a few large-insert BAC libraries are available to date for insect species, especially lepidopteran insects [10,11,14-17]. This could slow progress for the comprehensive molecular and genomics research of these clades.

Moths and butterflies, members of the insect order Lepidoptera, are the second most diverse group of animals, with at least 150,000 named species [18]. They are widespread members of the ecosystem, playing important roles as pollinators and prey, and are among the most destructive agricultural pests. Clearly, Lepidoptera are under-represented in terms of genomic resources and knowledge relative to their biological and economic status. This research was designed mainly to construct comprehensive BAC library resources for two species of moths, the tobacco hornworm, *Manduca sexta *and the tobacco budworm, *Heliothis virescens*, and one species of butterfly, the Müllerian mimic, *Heliconius erato*. These species have genome sizes ranging from 400 to 500 Mb/haploid genome (395 Mb for *H. erato *[19], 404 Mb for *H. virescens *[20], and 500 Mb for *M. sexta *[J. S. Johnston, pers. communication]) and are widely-used models for studying fundamental problems in neurobiology [21], olfaction [22], development [23], and immune responses [24] (*M. sexta*]; host feeding preferences [25] and evolution of insecticide resistance [26] and sexual communication systems [27] (*H. virescens*); and wing pattern mimicry [(*H. erato*) [28]. Moths and butterflies are estimated to have diverged from each other at least 50–60 million years ago [18]. The sphingid, *M. sexta*, is a member of the same superfamily, Bombycoidea, as the domesticated silkworm, *Bombyx mori*, the current genome model for Lepidoptera [8,9], and the noctuid, *H. virescens*, is related to other pest noctuids currently being used for genomic studies including *Spodoptera frugiperda *[16,29] and *Helicoverpa armigera *[30]. Here, we report the construction and characterization of six large-insert BAC libraries for these species and the first insight into the constitution and evolution of their genomes. The libraries will enable a large community of scientists to isolate and study the genes controlling these processes, provide new tools for lepidopteran systematics, and serve as critical resources for comparative genomic studies and genome sequencing of this important group of organisms.

## Results

### Development of procedures for preparation of high-molecular-weight (HMW) DNA

One of the most important steps toward construction of high-quality BAC libraries is preparation of high-quality megabase DNA. Since no procedure was available for preparation of HMW DNA from these insects, we first developed a method for megabase DNA preparation by testing different DNA isolation buffer systems and tissues collected at different developmental stages of the insects. The results showed that the day-10 pupae (males and females) of *M. sexta *and day-4 pupae (males and females) of *H. virescens *and *H. erato *were most suitable for megabase DNA isolation using a buffer system containing 0.1 M NaCl, 10 mM Tris-HCl, 10 mM EDTA, pH 9.4, and 0.15% β-mercaptoethanol. The DNA isolated with this method was not only large in size (> 1000 kb), but also readily digestible and clonable, thus being well-suited for BAC library construction.

### Library construction

**Figure 1:**
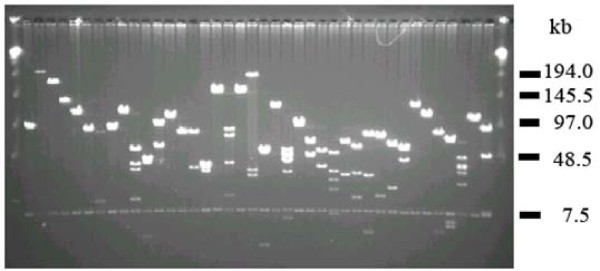
**BAC clones randomly selected from the *M. sexta Bam *HI (MSB) BAC library and analyzed by pulsed-field gel electrophoresis**. BAC DNA was isolated, digested with *Not*I to release the insert DNA from the cloning vector, run on a 1% agarose gel and stained with ethidium bromide. The average insert size of the clones was estimated to be 150 kb. The common band (7.5 kb) appearing in all BAC lanes is from the pEBAC1 cloning vector. Outer lanes are Lambda ladder PFG marker (New England BioLabs, USA).

The major goal of this study was to develop BAC resources that are widely usable for molecular and genomic studies of the insects, including whole genome physical mapping and sequencing. Therefore, we constructed two BAC libraries for each species with *Bam*HI and *Eco*RI in the BAC vector pECBAC1. Table 1 summarizes the characteristics of the six BAC libraries constructed. The libraries were named MSB and MSR for *M. sexta *(MS) and B or R for *Bam*HI or *Eco*RI, respectively, HVB and HVR for *H. virescens*, and HEB and HER for *H. erato*. The insert sizes of the library clones were estimated based on a random sample of 200–300 BACs from each library digested with *Not*I, a relatively rare cutter in lepidopteran DNA, and fractionated on pulsed-field gels. A typical pulsed field gel pattern for a set of random clones selected from the MSB BAC library is shown in Figure 1. The average insert sizes of the libraries ranged from 150–175 kb, and the proportion of the insert-empty clones was <5%. Each library contained from 19,200 to 21,504 clones which were arrayed into 384-well microtiter plates. Based on the number of clones and average insert sizes of each library, we estimated that the genome coverage of each library ranged from 6 ×–8 × genome equivalents, with the two combined libraries of each species having a genome coverage of 13.0 ×–16.3 × (Table 1). Based on this and previous studies [1,2,6,7,13], these BAC library resources should be well-suited for many kinds of molecular and genomic research, including whole genome physical mapping and sequencing.

**Table 1:** Basic parameters of lepidopteran BAC libraries

Library name	Species	Cloning enzyme	Mean insert size (kb)	Rate of insert-empty clones	No. of clones arrayed	No. of plates arrayed	Estimated genome coverage	Genome coverage of combined libraries
HEB	*H. erato*	*Bam*HI	175	<5%	19,200	50	8.1X	15.4X
HER	*H. erato*	*Eco*RI	153	<2%	19,200	50	7.3X	
HVB	*H. virescens*	*Bam*HI	171	<2%	21,504	56	9.0X	16.3X
HVR	*H. virescens*	*Eco*RI	150	1%	19,584	51	7.3X	
MSB	*M. sexta*	*Bam*HI	152	<1%	19,968	52	6.0X	13.0X
MSR	*M. sexta*	*Eco*RI	165	<1%	21,504	56	7.0X	

### BAC library screening

As an independent test of genome coverage, to demonstrate the utility of the libraries, and to isolate BACs containing genes of importance, the libraries were robotically double-spotted onto Nylon membrane filters in 3 × 3 format and screened with gene sequence-specific probes that are of interest for studies of the lepidopteran models. These included genes involved in olfaction (*MsOR1*, *MsOR3*, and *HvHR16*), nerve axon growth and guidance (*MsNos128*, *MsEph*, *MsFasII*, and *MsPlexA*), hormone action (*MsE75*, and *HvPTTH*), wing patterning (*Hewg*, *Heptc*, and *HeCi*), Bt toxin action (*HvAPN120 *and *Hvcad*), and ribosomal protein structure (*HvRpS4*, *HeRpS5*, *HeRpS9*, *HeRpL3*, and *HeRpL10*), some of which have also served as anchor loci for comparative linkage mapping [5,31]. Tables 2, 3 and 4 summarize the library screening results. Although the number of hits for individual probes varied widely, which may reflect the uneven distribution of the clones constructed with a single enzyme, the average number of hits in a library was close to the expected genome coverage estimated by the library insert sizes and the genome size (Table 1). The library genome coverage estimated by hybridization was slightly higher than the values expected by the BAC library insert sizes and the genome DNA content for the libraries constructed for *H. virescens *(16.7 hits per probe vs. 16.3 ×), and slightly lower for the libraries from *M. sexta *(12.3 hits per probe vs. 13.0 ×) and *H. erato *(13.9 hits per probe vs. 15.4 ×).

**Table 2:** Number of positive clones on the *Manduca sexta *library filters.

	Library	MSR 7.0X	MSB 6.0X	Expected 13.0X	
	
Probe	Function	Hits per library	Hits per library	Total hits	Probe source
Eph	eph-ephrin signaling	8	4	12	A. Nighorn
Nos 128	nitric oxide signal transduction	6	3	9	A. Nighorn
MsOR1	olfactory receptor	3	4	7	H. Robertson
MsOR3	olfactory receptor	11	11	22	H. Robertson
Fasciclin II	neuronal cell recognition	12	3	15	P. Copenhaver
Plexin A	axon guidance	10	--	10	P. Copenhaver
E75 Exon 4/5	ecdysone receptor	8	3	11	L. Riddiford

	Ave. no. hits per probe	8.3	4.7	12.3	

Probes were derived from bacterial plasmids.

**Table 3:** Number of positive clones on *Heliothis virescens *library filters.

Library		HVR 7.3X	HVB 9.0X	Expected 16.3X	
	
Probe	Function	Hits per library	Hits per library	Total Hits	Probe source
K390	cap binding protein	7	14.8	22	R. Palli
K386°	ribosomal protein S4	10	1.1	11	R. Palli
L086°	TIA-1	5	5.4	10	R. Palli
APN120°	Midgut receptor	8	3	11	S. Gill
Cadherin-650	Bt receptor	5.8	4.4	10	L. Gahan
Cadherin-1 kb	Bt receptor	3	--	3	S. Gill
HR 16	Olfactory receptor	--	17	17	F. Gould
PTTH	peptide hormone	--	18	18	PCR/GenBank

	Ave. no. hits per probe	7.6	9.1	16.7	

Probes were derived from bacterial plasmids except for PTTH. Decimals in hits reflect extrapolation for filters that were not screened.

**Table 4:** Number of positive clones on *Heliconius erato *library filters.

Library		HER 7.3X	HEB 8.1X	Expected 15.4X	
	
Probe	Function	Hits per library	Hits per library	Total hits	Probe source
RpS5	ribosomal protein S5	2	5	7	O. McMillan
RpS9	ribosomal protein S9	4	5	9	O. McMillan
RpL3	ribosomal protein L3	10	1	11	O. McMillan
RpL10	ribosomal protein L10	1	3	4	O. McMillan
Wingless	Signal transduction	13	12	25	O. McMillan
Ef1-α	Translation elongation factor	13	8	21	PCR/Genbank

	Ave. no. hits per probe	7.2	5.7	13.9	

Probes were derived from PCR products amplified from genomic DNA.

### BAC end sequences (BESs)

To validate the libraries further, estimate levels of contamination by microbial or organellar sequences and obtain some information about the constitution of the insect genomes, one 96-well plate per library, thus two 96-well plates per species, was sequenced from both ends of each clone. A total of 246–299 BESs were successfully generated for each species (Table 5). The sequences had an average read length of 630 nucleotides, with an average of 560 Q20 nucleotides. Analysis of the BESs indicated that there was no evidence of contamination with DNA from organelles, the *E. coli *host, or other microbes that were potentially carried by the DNA source insects. The sequences are registered in the trace archives of GenBank described in Methods [see Additional file 1].

**Table 5:** Analysis of the BESs of *H. erato *(HEB and HER), *H. virescens *(HVB and HVR), and *M. sexta *(MSB and MSR) using the RepeatMasker program [47].

BAC library	Total BAC ends sequenced	No. BAC ends with sequences	Successful rate of BAC end sequencing (%)	Average length Q20 sequences (bp)	Total length Q20 sequences (bp)
HEB/HER	384	246	64.06	478	117702
HVB/HVR	384	299	77.86	665	198779
MSB/MSR	384	273	71.09	659	180031

		All repeats		Retroelements	
BAC library	GC content (%)	Length (bp)/no.	Rate in genome (%)	Length (bp)/no.	Rate in genome (%)

HEB/HER	32.35	11781/231	10.01	777/2	0.66
HVB/HVR	36.18	6287/103	3.16	1178/3	0.59
MSB/MSR	35.19	6018/125	3.34	1490/10	0.83

	DNA transposons		Rolling circles		Small RNA
BAC library	Length (bp)/no.	Rate in genome (%)	Length (bp)/no.	Rate in genome (%)	Length (bp)/no.

HEB/HER	279/2	0.24	701/1	0.6	0
HVB/HVR	0	0	0	0	1331/2
MSB/MSR	535/1	0.3	0	0	166/1

	Small RNA	Simple repeats		Low complex repeats	
BAC library	Rate in genome (%)	Length (bp)/no.	Rate in genome (%)	Length (bp)/no.	Rate in genome (%)

HEB/HER	0	777/17	0.66	9247/206	7.86
HVB/HVR	0.67	779/21	0.39	2999/79	1.51
MSB/MSR	0.09	487/11	0.27	3340/103	1.86

### The GC and repeat element contents of the insect genomes

To provide a first insight into the constitution of the insect genomes, we analyzed the BESs using the RepeatMasker program, with an emphasis on the contents of GC and repeat elements including retroelements, DNA transposons, simple repeats, and low complexity repeats (Table 5 [see Additional files 2, 3 and 4]). The GC contents of the genomes ranged from 32.35% (*H. erato*) to 36.18% (*H. virescens*), with the genome of the butterfly having 3.34% less GC than those of the moths. A total of 117.702 kb of BESs was obtained from the genome of *H. erato*. The sequence contained a total of 231 repeats, which is equivalent to 10.01% of the genome. Two hundred six of these repeats were categorized as low complexity (7.86% of the genome). This number contrasted with those of *H. virescens *and *M. sexta*. *H. virescens *had a total of 198.779 kb BESs from which a total of 103 repeats (3.16% of the genome) were identified, of which 79 were categorized as low complexity (1.51% of the genome). Similarly, a total of 180.031 kb of BESs generated from the *M. sexta *BACs were found to contain a total of 125 repeats (3.34% of the genome), of which 103 were categorized as low complexity (1.86% of the genome). Therefore, 3.16–10.01% of the lepidopteran genomes comprised repeat elements, of which a majority was categorized as low complexity repeats. The overall percentage of repeat elements was approximately 3-fold larger for the butterfly than for the moth genomes; further, the percentage of the low complexity repeats was > 4-fold larger for the butterfly than for the moth genomes. Among the low complexity repeats, 11 were longer than 100 bp (244 bp for the largest low complexity repeat), all of which were obtained from *H. erato*, whereas all remaining low complexity repeats obtained from *M. sexta *and *H. virescens *were shorter than 100 bp.

Retrotransposons, transposons and simple repeats were also identified in the BESs, but altogether they comprised <1% of the genomes. Nevertheless, the percentage of simple sequence repeats in the butterfly genome (0.66%) was about 2-fold higher than those of the moth genomes (0.39% and 0.27%). Moreover, a total of 15 retro-elements were identified in the BESs of all three species whereas only 3 DNA transposons were identified, suggesting that retro-elements are generally more abundant than DNA transposons in these genomes.

### BLAST hits of the BESs

Using the discontiguous Megablast program to query the database of all organisms available in GenBank, we searched for matches to BESs of the three species by BLASTn after masking with the RepeatMasker program (Tables 6 and 7 [see Additional files 5, 6 and 7]). One hundred eight (43.9%) of the 246 *H. erato *BESs had a total of 1,364 hits to GenBank sequences, with an average of 12.6 hits per BES (range 1–74 hits) at the default e-value of 3.0–3.5E–140. Of the 299 *H. virescens *BESs, 134 (44.8%) had a total of 1,128 hits, with each BES having an average of 8.4 hits (range 1–69 hits). Of the 273 *M. sexta *BESs, 127 (34.1%) had a total of 1,516 hits, with each BES having an average of 11.9 hits (range 1–57 hits; Table 6).

**Table 6:** BLASTn hits of the BESs of *H. erato *(HEB and HER), *H. virescens *(HVB and HVR), and *M. sexta *(MSB and MSR) against the sequences of all organisms available in GenBank (June 2007) calculated by number of sequences.

	Leps	Bm	Hm	He	Hv	Ms	Other insects	Animals
HEB/HER	1174	23	1059	16	5	1	52	122
HVB/HVR	793	264	53	2	48	24	66	143
MSB/MSR	459	41	3	0	1	340	81	323

	Human	Mouse	Zebrafish	Viruses/bacteria	Fungi	Plants	Insect total	Total

HEB/HER	14	23	10	4	2	10	1226	1364
HVB/HVR	32	29	23	13	8	6	859	1029
MSB/MSR	81	90	63	10	10	50	530	933

Bm, *Bombyx mori*; He, *Heliconius erato*; Hm, *H. melpomene*; Hv, *Heliothis virescens*; and Ms, *Manduca sexta*.

**Table 7:** BLASTn hits of the BESs of *H. erato *(HEB and HER), *H. virescens *(HVB and HVR), and *M. sexta *(MSB and MSR) against the sequences of all organisms available in GenBank (June 2007) calculated by number of species.

	Leps	Other insects	Animals	Viruses/bacteria	Fungi	Plants	Total
HEB/HER	27	11	28	3	3	4	76
HVB/HVR	41	11	22	9	6	3	92
MSB/MSR	24	6	28	9	7	7	81

We further examined the distribution of the BLASTn hits of the BESs among different species (Tables 6 and 7 [see Additional files 5, 6 and 7]). Of the 1,364 *H. erato *hits, 1,226 (89.9%) were from 38 insect species. The majority of the latter hits were from other *Heliconius *species, including *H. melpomene*, *H. doris*, *H. himera*, *H. erato*, and *H. cydno*, whereas the remaining hits were from *H. virescens*, *Helicoverpa armigera *(cotton bollworm), and *H. zea *(corn earworm). Exclusively, 1,059 (77.6%) of the 1,364 *H. erato *hits belonged to *H. melpomene*. Moreover, we found that both ends of three *H. erato *BACs, LQCBT56 (HER08E08), LQCBU02 (HEB02A01), and LQCBU16 (HEB04B01), had more than 10 discontiguous homologous sequences each to *H. melpomene *BACs registered in GenBank. These results suggested that this set of BACs may represent homologous regions. Complete sequencing of the three *H. erato *BACs will provide more information about the extent of microsynteny and evolution between these two species.

In comparison, 859 (83.5%) of the 1,029 hits for *H. virescens *BESs were from 52 insect species (Table 7). The top four values for BES hits included sequences from *B. mori*, *H. melpomene*, *H. virescens*, and *M. sexta*. Compared with 27 and 24 lepidopteran species with sequences hit by the *H. erato *and *M. sexta *BESs, respectively, 41 lepidopteran species were represented in sequences hit by the *H. virescens *BESs, even though similar numbers of BESs (246–299) were queried for the three species. This suggested that the *H. virescens *genome is a broader representative of Lepidoptera than *H. erato *or *M. sexta*.

Of the 933 hits for *M. sexta *BESs, 530 (56.8%) were from 30 insect species, including 340 hits (36.4%) exclusive to known sequences in *M. sexta*. Moreover, the highest numbers of *M. sexta *hits included sequences from vertebrates, including mouse (90), human (81), and zebrafish (63), and plants (50). By comparison to the genomic sequences of *H. erato *and *H. virescens*, the *M. sexta *genome seems to have evolved to be less unique as a lepidopteran, showing more similarities to the genomic sequences of other animals and even plants.

It was also found that most of the homologous sequences were shorter than 200 bp. However, large contiguous homologous sequences (>200 bp) were found to be associated with lepidopteran genes encoding proteins involved in hormone metabolism, structural proteins, and metabolic enzymes [see Additional files 5, 6 and 7]. An independent search of the BESs using BLASTx in GenBank and ButterflyBase [32] yielded an average of 10.7 hits per species with high homology to confirmed coding regions of identified genes at e-values less than 1E-10 and bitscores in a range of 55–383 [see Additional file 8]. Additional hits were to ORFs with high similarity to features associated with retrotransposons, such as reverse transcriptase, gag-pol polyprotein, and endonuclease [33] and non-LTR transposons found in the silkworm genome, such as TRAS and SART [34]. Due to the limited sequencing information, the BLASTn results and discovery of putative genes are presented as potential features to be confirmed by further analysis.

## Discussion

We have constructed six BAC libraries for three lepidopteran model species (2 moths and 1 butterfly). These libraries not only have large-insert sizes (150 – 175 kb) and deep genome coverage (13 × – 17 ×), but also have a low level of insert-empty clones (<5%) and no detected contamination with DNA from organelles and microbes potentially living on the source insects, as indicated by BES analysis. Moreover, the genome coverage and quality of the libraries have been verified independently by screening high-density filters of the libraries with a set of single-copy genes or ESTs. The observation that none of the libraries was contaminated with microbial DNA potentially carried by the source insects was expected, because the self-contained non-feeding pupal stage used as a DNA source for the library construction had purged their guts at the end of larval development. However, we did observe 6, 21 and 20 short sequences in the He, Hv and Ms BESs, respectively, which were homologous to viral, bacterial, and fungal sequences (Tables 6 and 7 [see Additional files 5, 6 and 7]). We believe that the homologues are real, but not from sample contamination because they sit in the middle of BESs. These results perhaps provide a line of preliminary evidence for the presence of microbial sequences in these lepidopteran genomes, possibly by horizontal transfer. Similar findings have been obtained in *B. mori *[35]. On the other hand, considering the small fraction (~0.5%) of the BAC libraries sampled, a more direct test of organelle contamination could be accomplished by using mitochondrial sequences as probes for hybridization. Furthermore, since the libraries of each species were constructed with two restriction enzymes (*Eco*RI and *Bam*HI) complementary in the GC content of their restriction sites, the genome coverage should be much better distributed along the genome than those constructed with a single enzyme [13,36]. Therefore, these libraries could provide useful resources for comprehensive genomics research of the three model lepidopterans.

The libraries, library filters and individual clones have been distributed to a number of laboratories and are presently being used for following studies: 1) walking to wing colour patterning genes from closely linked AFLP sequences in *H. erato *[17,37]; 2) testing synteny between *M. sexta*, *B. mori*, and *H. melpomene *by chromosomal fluorescence *in situ *hybridization using BACs containing orthologous genes as probes [5,38]; 3) analysis of full-length coding and regulatory regions for the *M. sexta Broad *gene (L. Riddiford, personal communication); and 4) analysis of *H. virescens HR16 *putative odor receptor sequences (F. Gould, personal communication).

The results of this study (Tables 5, 6 and 7) have provided a snapshot of the basic characters of the genomes of a group of ditrysian moths and butterflies which diverged from each other at least 50–60 million years ago [18]. First, the genomes of all three species are AT-rich (64–68%), with the genome of the butterfly (*H. erato*) having an AT content more than 3% higher than those of the moths (*M. sexta and H. virescens*). Second, the results show that all three insect genomes contain relatively small fractions of repeat elements (3–10%), including retro-transposons, transposons, simple repeats, and low complexity repeats. These results are in agreement with the small genomes of the species (400–500 Mb/1C) which generally tend to contain smaller fractions of repeat elements. Of these three insect species, the butterfly genome contains 3–5-fold more repeat elements (10.01% all repeats), especially low complexity repeats, than the two moth genomes. Papa reported that the total repetitive sequences accounted for about 26% of the genomic regions linked to wing pattern variation in *H. erato *[37]. The difference could be an effect of more *H. erato*-specific repeats documented, sampling of a specific region with a higher average repeat density, or both. Third, whereas the three insect genomes all contain a small number (<1%) of retro-elements, DNA transposons and simple repeats, retro-elements seem much more abundant than DNA transposons, and the butterfly genome is two-fold richer in simple repeats than the two moth genomes. Compared with published information from *B. mori*, the finding of such a low percentage of repeat contents in these three lepidopteran species is surprising, especially for *M. sexta*, which is in the same superfamily as the silkworm, Bombycoidea. Xia et al. [9] estimated about 20% of the *B. mori *genome to be composed of "transposable elements;" further, early work based on Cot hybridization kinetics estimated about 45% of the silkworm genome to be composed of repetitive sequences [39]. More recently Osanai-Futahashi et al. reported that the TEs made up 35% of the silkworm genome and contributed greatly to the genome size [40]. One may argue that we might simply have not identified all the relevant repeats in the BESs, but our argument is supported by the following evidence. The genome of the butterfly, *H. erato*, contains extremely large numbers (1059 of 1364 hits) of small duplicated sequences or "novel repeats" (not registered in GenBank) which are homologous to three completely sequenced BAC clones (118 kb of AEHM-41C10, 112 kb of AEHM-46M10, and 118 kb of AEHM-7G12) of *H. melpomene*. This in turn indicates the presence of novel repetitive or duplicated sequences in the *H. melpomene *genome [see Additional file 5]. Large-scale end sequencing of the complete BAC libraries will uncover more detailed aspects of these butterfly and moth genomes, and provide more information for fundamental studies of lepidopteran insects in general.

The BLAST analysis of the sampled BESs has also provided insights into the evolution of these insect genomes. It is not surprising to find the top hits are to the sequences of lepidopteran species, but it is quite surprising that the highest numbers of *M. sexta *BES hits were to the sequences of other animals and plants rather than to *B. mori *(Tables 6 and 7). This finding suggests that although all the genomes have undergone changes since the split from the most recent common ancestor, they may have done so along different trajectories, with the *M. sexta *genome retaining some sequences in common with plants and animals that have been either lost or modified to a greater extent in *H. virescens *and *H. erato*. Such a hypothesis can only be tested when more genomic data are available for these lepidopteran insects. Moreover, the BESs of the butterfly (*H. erato*) are well-matched only to the sequences of *H. melpomene*. This suggests that not only is the butterfly more related to *H. melpomene *than to the two moth species, as expected, but this group has also diverged to a greater extent, resulting in a higher level of species- or evolutionary lineage-specific sequences. This argument is further supported by the finding that 27 of the 76 species having sequence matches to the BESs of *H. erato *(35.5%) were from other Lepidoptera. This number is 6% higher than that of *M. sexta *but 9% less than that of *H. virescens*. By contrast, the total number of top hits to lepidopteran species for *H. virescens *BESs was 41, or 17 and 14 more than for *M. sexta *and *H. erato*, respectively. Therefore, the genome of *H. virescens *may be a better representative of the genomes of Lepidoptera as a whole (Table 7).

One may argue that the RepeatMasker program might not mask the repeat sequences completely because of the limited amount of repeat elements available in the public database; however, this does not appear to have affected the BLAST results significantly. For instance, *B. mori *represents the species having the most sequence information in GenBank among the lepidopteran species; however, we found significantly different hits, 23, 264 and 41 for the BESs of *H. erato*, *H. virescens*, and *M. sexta*, respectively (Table 6). Moreover, there are many more *Drosophila *spp. sequences in GenBank than for any other insect; however, we only observed limited numbers of *Drosophila *sequence hits: 25, 16 and 36 for the BESs of *H. erato*, *H. virescens*, and *M. sexta*, respectively (not shown); there were no large (≥ 200 bp) hits for any *Drosophila *sequence, and only one large (≥ 200 bp) hit each for *Apis mellifera *(honey bee), for the BESs of *H. virescens *and *M. sexta*, even though the honey bee genome is also fully sequenced [see Additional files 5, 6 and 7]. Similarly, a large number of top BLASTx hits were to protein sequences in lepidopteran species (12/32) or other insects (17/32), such as *Acyrthosiphon pisum *(pea aphid) and *Tribolium castaneum *(red flour beetle), of which relatively few were to *Drosophila spp *(3/32).

## Conclusion

We constructed six high-quality, deep-genome coverage BAC libraries, two libraries for each of three lepidopteran model species: *H. erato*, *H. virescens*, and *M. sexta*, with two restriction enzymes, respectively. As the average clone insert size of the libraries ranging from 152–175 kb, we estimated that the genome coverage of each library ranged from 6–9 ×, with the two combined libraries of each species being equivalent to 13.0–16.3 × haploid genomes. This genome coverage should be sufficient for many aspects, if not all, of genomics studies of each species, including genome-wide physical mapping and genome sequencing.

Genomic sequence sample analysis of the moths and butterfly has provided an initial insight into the constitution and evolution of their genomes. Although large-scale genome sequencing is needed to further decipher the genomes of the species, especially their gene contents, the basic characteristics of the repeated sequence portion of each genome is useful information for our understanding of the genomes and their evolution. The high-quality BAC libraries of the insects, together with the gene-containing BACs and BAC end sequences, provide valuable information, resources and tools for comprehensive studies of the insect genomes and for addressing many fundamental questions in Lepidoptera.

## Methods

### Insect Materials

#### Source of DNA

To minimize the potential polymorphism of the source DNA for BAC library construction, we sought insects that were as inbred as possible. For each species, we used progeny from a single pair mating to restrict the potential polymorphism of the insects to a maximum of 4 alleles per locus. Because the source strains were at least partially inbred, we expected significantly less polymorphism at many loci. This strategy also minimized the number of haplotypes in a library, since intra-chromosomal exchange (crossing over) occurs only in lepidopteran males. The source of DNA for *M. sexta *was a colony that was maintained without outcrossing for at least 30 years (L. Riddiford, U. Washington). For *H. virescens *(F. Gould, North Carolina State U.) and *H. erato *(O. McMillan, U. Puerto Rico), the DNA source was a colony that had to be replenished periodically from wild populations to avoid inbreeding depression. Whereas *M. sexta *and *H. virescens *deposit large numbers of eggs in a short time, enabling a relatively synchronous rearing, *H. erato *lays only a few eggs each day for several months. Therefore, we collected and froze insects at an appropriate stage (day-4 pupae) based on pilot studies. Consequently, it took more than 6 months to accumulate a sufficient number of animals (~200) to prepare high molecular weight (HMW) DNA for library construction of this species.

#### Vouchering

To maintain the identity of the DNA source animals, voucher specimens from the same families used for BAC library construction were archived at the Museum of Comparative Zoology, Harvard University (*M. sexta*, N. Pierce), and at the North Carolina State University Insect Collection (*H. virescens *and *H. erato*, F. Gould). The archived specimens included dried adult wings and the corresponding bodies preserved in 70–100% alcohol at -20°C or -80°C.

### BAC library construction

The pECBAC1 vector [41] was used in the library construction [42]. Vector DNA was isolated by the alkaline lysis method, purified by cesium chloride gradient centrifugation, digested completely with either *Bam*HI or *EcoR*I, and dephosphorylated with calf intestinal alkaline phosphatase. The digested vector DNA was precipitated, dissolved in TE (10 mM TrisHCl, 1 mM EDTA, pH 8.0), adjusted to 10 ng/μl, and stored at -20°C before use [1,2,13,43].

HMW DNA was isolated from the insects using frozen pupal tissues and buffer system (see Results) according to the procedure described by Wu et al. [14]. The BAC libraries were constructed using an improved procedure developed in our laboratory [1,2,13,43]. Briefly, HMW genomic DNA plugs were prepared from day-10 pupae (males and females) of *M. sexta *and day-4 pupae (males and females) of *H. virescens *and *H. erato*. DNA was partially digested with *Bam*HI or *Eco*RI, size-fractionated in a clamped homogeneous electrical field (CHEF) apparatus (Bio-Rad), recovered by electroelution, and then ligated into the *Bam*HI or *EcoR*I site of the pECBAC1 vector, respectively. The ligated DNA was transformed into *E. coli *DH10B cells (Invitrogen, USA) by electroporation. The transformed cells were incubated in SOC medium with shaking at 250 rpm, 37°C for 1 h. Recombinant transformants were selected and incubated for 32 h at 37°C on LB agar (Invitrogen, USA) plates containing 12.5 μg/ml chloramphenicol, 0.5 mM IPTG, and 50 μg/ml X-gal.

### Insert size analysis and BAC library arraying

White colonies were randomly selected and grown in LB medium (Invitrogen, USA). BAC DNA was isolated, digested with *Not*I, and subjected to CHEF gel electrophoresis. The ligation that gave a transformation efficiency of 200 or more white colonies/μl ligation and that generated clones with the largest inserts was selected for library construction. The BAC colonies were manually arrayed as individual clones in 384-well microtiter plates containing 50 μl LB plus freezing broth with 12.5 μg/ml chloramphenicol [1,2,13,43,44]. After incubation at 37°C for 14 h, the microtiter plates were stored at -80°C. To facilitate their accessibility, all six BAC libraries have been made available to the public at the TAMU GENE *finder *Genomic Resources Center directed by H.-B. Z.

### BAC library screening

A GeneTAC G3 robotic workstation (Genomic Solutions, Inc., USA) was used to double-spot the BAC libraries onto 8 × 12-cm Hybond N+ filters (Amersham-Pharmacia, USA) in 3 × 3 format so that each high-density clone filter contained two spots of each clone from four 384-well microtiter plates (1,536 × 2 spots). The filters were processed according to Zhang [43] and Zhang et al. [44]. Filter screening was carried out using a non-radioactive detection system (ECL, Pharmacia Amersham/Pharmacia, USA) with X-ray film (Hyperfilm; Amersham/Pharmacia, USA) according to the manufacturer's instructions. All probes used except for putative olfactory receptors (MsOR1, MsOR3, and HvHR16) were verified as single copy by BLASTn search of KAIKObase [45] or BLASTx search of FlyBase [46] to confirm the presence of one chromosomal locus. The amount of hybridizing DNA per filter was adjusted to a range of 30–60 ng per filter based on the intensity of signal obtained after initial screening. We routinely used 0.5 M NaCl in the hybridization buffer, but in some cases increased stringency to 0.4 M to reduce background. We re-used filters without stripping until the background became too high to read positive signals reliably or until we detected carry-through; then we treated the filters to remove the probe DNA according to the manufacturer's instructions. Probe DNA was obtained from a variety of sources, including bacterial plasmids containing well-characterized cDNA sequences and PCR fragments amplified from genomic DNA based on sequences registered in GenBank. Insert DNA was amplified by PCR using primers designed from the plasmid vectors or within the insert sequence and purified on Wizard Spin columns (Promega, USA) before labelling with the ECL reagents. Although we were able to detect positive signals on filters hybridized with probes as short as 350–400 bp, probes of 1 kb or greater gave more consistent signal-to-noise ratios.

### BAC end sequencing and analysis

Ninety-six clones were randomly selected from each of the six BAC libraries and re-arrayed into a 96-well plate. Both ends of each clone were sequenced using the primer 5'-TAATACGACTCACTATAGGG-3' for the T7 end and 5'-GTTTTTTGCGATCTGCCGTTTC-3' for the SP6 end using the procedure developed at The Institute for Genomic Research [12] [see Additional file 1 for the original library clone names and the corresponding TIGR names]. The sequences are registered in the trace archives of GenBank under the following link:  SPECIES_CODE='HELICONIUS ERATO' AND CENTER_NAME='TIGR' or TI#: 908600791-908601036; SPECIES_CODE='HELIOTHIS VIRESCENS' AND CENTER_NAME='TIGR' or TI#: 908601037–908601335; and SPECIES_CODE='MANDUCA SEXTA' AND CENTER_NAME='TIGR' or TI#: 908601336–908601608. The resultant BESs were analyzed by utilizing the RepeatMasker program [47]. Finally, the masked BESs were BLASTed against the databases of all organisms by using the discontiguous megablast program at NCBI using the default criteria.

BLASTx searches were carried out against non-redundant protein sequences in GenBank (December 2008) and ButterflyBase version 2.92 [32]. Hits with e-values less than 1E-7 and bitscores greater than 50 were evaluated for similarity to coding regions of identified proteins and retrotransposons. High matches of similar sequences in more than one species was used as a criterion for provisional identification of a *bona fide *protein.

## Abbreviations

BAC: Bacterial Artificial Chromosome; BES: BAC end sequence; ORF: open reading frame.

## Authors' contributions

CW participated in the study design, BAC library construction, BAC end sequence data analysis and manuscript preparation. DP, DC and EN participated in the library screening. FS supported the BAC library assembly and filter preparation. SZ conducted the BAC end sequencing. H-BZ participated in the study design, BAC library construction and manuscript preparation. MRG participated in the study design, insect vouchering, library screening, BLASTx search and manuscript preparation. All authors read and approved the final manuscript.

## Supplementary Material

Additional file 1 **Correspondence of TIGR BAC end sequence names with their library clone names**. Table S1 lists TIGR BAC end sequence names and corresponding BAC library clone names. Click here for file

Additional file 2 **Classes of repeats in BESs of 3 lepidopteran species analyzed with the RepeatMasker tool**. Table S2 includes summaries of types of repeated sequences identified in the BESs of *H. erato, H. virescens*, and *M. sexta *using RepeatMasker. Click here for file

Additional file 3 **Lists of masked Lepidoptera repeats**. Table S3 lists the masked repeats in the BESs of *H. erato*, *H. virescens*, and *M. sexta*, their sequence characteristics, and location in the BESs. Click here for file

Additional file 4 **Detailed repeat element contents in the BESs of three lepidopteran models**. Table S4 summarizes the types of repeat elements found in the BESs of *H. erato*, *H. virescens*, and *M. sexta*. Click here for file

Additional file 5 ***Heliconius erato *BES BLASTn hits**. Table S5 lists the sequences hit by BLASTn search with *H. erato *BESs and their characteristics. Click here for file

Additional file 6 ***Heliothis virescens *BES BLASTn hits**. Table S6 lists the sequences hit by BLASTn search with *H. virescens *BESs and their characteristics. Click here for file

Additional file 7 ***Manduca sexta *BES BLASTn hits**. Table S7 lists the sequences hit by BLASTn search with *M. sexta *BESs and their characteristics. Click here for file

Additional file 8 **High scoring BES hits identified by BLASTx search**. Table S8 lists the high scoring BLASTx hits in ButterflyBase and GenBank for *H. erato, H. virescens*, and *M. sexta* Click here for file
