# Supplementary material for: Construction and sequence sampling of deep-coverage, large-insert BAC libraries for three model lepidopteran species
Source: BMC Genomics. 2009 Jun 26;10:283. doi: 10.1186/1471-2164-10-283 (PMC2718931; doi:10.1186/1471-2164-10-283)
Supplement: Additional file 1 — Correspondence of TIGR BAC end sequence names with their library clone names. Table S1 lists TIGR BAC end sequence names and corresponding BAC library clone names. [file 1471-2164-10-283-S1.doc]

Table S1-A. Correspondence of *Heliconius erato Bam*HI BAC end sequence names with their library clone names

| TIGR name | Library clone name_primer | TIGR name | Library clone name_primer |
| --- | --- | --- | --- |
| LQCBU01TFB | HEB01A01_M13F | LQCBU01TR | HEB01A01_M13R |
| LQCBU02TFB | HEB02A01_M13F | LQCBU02TR | HEB02A01_M13R |
| LQCBU03TFB | HEB03A01_M13F | LQCBU03TR | HEB03A01_M13R |
| LQCBU04TFB | HEB04A01_M13F | LQCBU04TR | HEB04A01_M13R |
| LQCBU05TFB | HEB05A01_M13F | LQCBU05TR | HEB05A01_M13R |
| LQCBU06TFB | HEB06A01_M13F | LQCBU06TR | HEB06A01_M13R |
| LQCBU07TFB | HEB07A01_M13F | LQCBU07TR | HEB07A01_M13R |
| LQCBU08TFB | HEB08A01_M13F | LQCBU08TR | HEB08A01_M13R |
| LQCBU09TFB | HEB09A01_M13F | LQCBU09TR | HEB09A01_M13R |
| LQCBU10TFB | HEB10A01_M13F | LQCBU10TR | HEB10A01_M13R |
| LQCBU11TFB | HEB11A01_M13F | LQCBU11TR | HEB11A01_M13R |
| LQCBU12TFB | HEB12A01_M13F | LQCBU12TR | HEB12A01_M13R |
| LQCBU13TFB | HEB01B01_M13F | LQCBU13TR | HEB01B01_M13R |
| LQCBU14TFB | HEB02B01_M13F | LQCBU14TR | HEB02B01_M13R |
| LQCBU15TFB | HEB03B01_M13F | LQCBU15TR | HEB03B01_M13R |
| LQCBU16TFB | HEB04B01_M13F | LQCBU16TR | HEB04B01_M13R |
| LQCBU17TFB | HEB05B01_M13F | LQCBU17TR | HEB05B01_M13R |
| LQCBU18TFB | HEB06B01_M13F | LQCBU18TR | HEB06B01_M13R |
| LQCBU19TFB | HEB07B01_M13F | LQCBU19TR | HEB07B01_M13R |
| LQCBU20TFB | HEB08B01_M13F | LQCBU20TR | HEB08B01_M13R |
| LQCBU21TFB | HEB09B01_M13F | LQCBU21TR | HEB09B01_M13R |
| LQCBU22TFB | HEB10B01_M13F | LQCBU22TR | HEB10B01_M13R |
| LQCBU23TFB | HEB11B01_M13F | LQCBU23TR | HEB11B01_M13R |
| LQCBU24TFB | HEB12B01_M13F | LQCBU24TR | HEB12B01_M13R |
| LQCBU25TFB | HEB01C01_M13F | LQCBU25TR | HEB01C01_M13R |
| LQCBU26TFB | HEB02C01_M13F | LQCBU26TR | HEB02C01_M13R |
| LQCBU27TFB | HEB03C01_M13F | LQCBU27TR | HEB03C01_M13R |
| LQCBU28TFB | HEB04C01_M13F | LQCBU28TR | HEB04C01_M13R |
| LQCBU29TFB | HEB05C01_M13F | LQCBU29TR | HEB05C01_M13R |
| LQCBU30TFB | HEB06C01_M13F | LQCBU30TR | HEB06C01_M13R |
| LQCBU31TFB | HEB07C01_M13F | LQCBU31TR | HEB07C01_M13R |
| LQCBU32TFB | HEB08C01_M13F | LQCBU32TR | HEB08C01_M13R |
| LQCBU33TFB | HEB09C01_M13F | LQCBU33TR | HEB09C01_M13R |
| LQCBU34TFB | HEB10C01_M13F | LQCBU34TR | HEB10C01_M13R |
| LQCBU35TFB | HEB11C01_M13F | LQCBU35TR | HEB11C01_M13R |
| LQCBU36TFB | HEB12C01_M13F | LQCBU36TR | HEB12C01_M13R |
| LQCBU37TFB | HEB01D01_M13F | LQCBU37TR | HEB01D01_M13R |
| LQCBU38TFB | HEB02D01_M13F | LQCBU38TR | HEB02D01_M13R |
| LQCBU39TFB | HEB03D01_M13F | LQCBU39TR | HEB03D01_M13R |
| LQCBU40TFB | HEB04D01_M13F | LQCBU40TR | HEB04D01_M13R |
| LQCBU41TFB | HEB05D01_M13F | LQCBU41TR | HEB05D01_M13R |
| LQCBU42TFB | HEB06D01_M13F | LQCBU42TR | HEB06D01_M13R |
| LQCBU43TFB | HEB07D01_M13F | LQCBU43TR | HEB07D01_M13R |
| LQCBU44TFB | HEB08D01_M13F | LQCBU44TR | HEB08D01_M13R |
| LQCBU45TFB | HEB09D01_M13F | LQCBU45TR | HEB09D01_M13R |
| LQCBU46TFB | HEB10D01_M13F | LQCBU46TR | HEB10D01_M13R |
| LQCBU47TFB | HEB11D01_M13F | LQCBU47TR | HEB11D01_M13R |
| LQCBU48TFB | HEB12D01_M13F | LQCBU48TR | HEB12D01_M13R |
| LQCBU49TFB | HEB01E01_M13F | LQCBU49TR | HEB01E01_M13R |
| LQCBU50TFB | HEB02E01_M13F | LQCBU50TR | HEB02E01_M13R |
| LQCBU51TFB | HEB03E01_M13F | LQCBU51TR | HEB03E01_M13R |
| LQCBU52TFB | HEB04E01_M13F | LQCBU52TR | HEB04E01_M13R |
| LQCBU53TFB | HEB05E01_M13F | LQCBU53TR | HEB05E01_M13R |
| LQCBU54TFB | HEB06E01_M13F | LQCBU54TR | HEB06E01_M13R |
| LQCBU55TFB | HEB07E01_M13F | LQCBU55TR | HEB07E01_M13R |
| LQCBU56TFB | HEB08E01_M13F | LQCBU56TR | HEB08E01_M13R |
| LQCBU57TFB | HEB09E01_M13F | LQCBU57TR | HEB09E01_M13R |
| LQCBU58TFB | HEB10E01_M13F | LQCBU58TR | HEB10E01_M13R |
| LQCBU59TFB | HEB11E01_M13F | LQCBU59TR | HEB11E01_M13R |
| LQCBU60TFB | HEB12E01_M13F | LQCBU60TR | HEB12E01_M13R |
| LQCBU61TFB | HEB01F01_M13F | LQCBU61TR | HEB01F01_M13R |
| LQCBU62TFB | HEB02F01_M13F | LQCBU62TR | HEB02F01_M13R |
| LQCBU63TFB | HEB03F01_M13F | LQCBU63TR | HEB03F01_M13R |
| LQCBU64TFB | HEB04F01_M13F | LQCBU64TR | HEB04F01_M13R |
| LQCBU65TFB | HEB05F01_M13F | LQCBU65TR | HEB05F01_M13R |
| LQCBU66TFB | HEB06F01_M13F | LQCBU66TR | HEB06F01_M13R |
| LQCBU67TFB | HEB07F01_M13F | LQCBU67TR | HEB07F01_M13R |
| LQCBU68TFB | HEB08F01_M13F | LQCBU68TR | HEB08F01_M13R |
| LQCBU69TFB | HEB09F01_M13F | LQCBU69TR | HEB09F01_M13R |
| LQCBU70TFB | HEB10F01_M13F | LQCBU70TR | HEB10F01_M13R |
| LQCBU71TFB | HEB11F01_M13F | LQCBU71TR | HEB11F01_M13R |
| LQCBU72TFB | HEB12F01_M13F | LQCBU72TR | HEB12F01_M13R |
| LQCBU73TFB | HEB01G01_M13F | LQCBU73TR | HEB01G01_M13R |
| LQCBU74TFB | HEB02G01_M13F | LQCBU74TR | HEB02G01_M13R |
| LQCBU75TFB | HEB03G01_M13F | LQCBU75TR | HEB03G01_M13R |
| LQCBU76TFB | HEB04G01_M13F | LQCBU76TR | HEB04G01_M13R |
| LQCBU77TFB | HEB05G01_M13F | LQCBU77TR | HEB05G01_M13R |
| LQCBU78TFB | HEB06G01_M13F | LQCBU78TR | HEB06G01_M13R |
| LQCBU79TFB | HEB07G01_M13F | LQCBU79TR | HEB07G01_M13R |
| LQCBU80TFB | HEB08G01_M13F | LQCBU80TR | HEB08G01_M13R |
| LQCBU81TFB | HEB09G01_M13F | LQCBU81TR | HEB09G01_M13R |
| LQCBU82TFB | HEB10G01_M13F | LQCBU82TR | HEB10G01_M13R |
| LQCBU83TFB | HEB11G01_M13F | LQCBU83TR | HEB11G01_M13R |
| LQCBU84TFB | HEB12G01_M13F | LQCBU84TR | HEB12G01_M13R |
| LQCBU85TFB | HEB01H01_M13F | LQCBU85TR | HEB01H01_M13R |
| LQCBU86TFB | HEB02H01_M13F | LQCBU86TR | HEB02H01_M13R |
| LQCBU87TFB | HEB03H01_M13F | LQCBU87TR | HEB03H01_M13R |
| LQCBU88TFB | HEB04H01_M13F | LQCBU88TR | HEB04H01_M13R |
| LQCBU89TFB | HEB05H01_M13F | LQCBU89TR | HEB05H01_M13R |
| LQCBU90TFB | HEB06H01_M13F | LQCBU90TR | HEB06H01_M13R |
| LQCBU91TFB | HEB07H01_M13F | LQCBU91TR | HEB07H01_M13R |
| LQCBU92TFB | HEB08H01_M13F | LQCBU92TR | HEB08H01_M13R |
| LQCBU93TFB | HEB09H01_M13F | LQCBU93TR | HEB09H01_M13R |
| LQCBU94TFB | HEB10H01_M13F | LQCBU94TR | HEB10H01_M13R |
| LQCBU95TFB | HEB11H01_M13F | LQCBU95TR | HEB11H01_M13R |
| LQCBU96TFB | HEB12H01_M13F | LQCBU96TR | HEB12H01_M13R |

Table S1-B. Correspondence of *Heliconius erato Eco*RI BAC end sequence names with their library clone names

| TIGR name | Library clone name_primer | TIGR name | Library clone name_primer |
| --- | --- | --- | --- |
| LQCBT01TFB | HER01A01_M13F | LQCBT01TR | HER01A01_M13R |
| LQCBT02TFB | HER02A02_M13F | LQCBT02TR | HER02A02_M13R |
| LQCBT03TFB | HER03A03_M13F | LQCBT03TR | HER03A03_M13R |
| LQCBT04TFB | HER04A04_M13F | LQCBT04TR | HER04A04_M13R |
| LQCBT05TFB | HER05A05_M13F | LQCBT05TR | HER05A05_M13R |
| LQCBT06TFB | HER06A06_M13F | LQCBT06TR | HER06A06_M13R |
| LQCBT07TFB | HER07A07_M13F | LQCBT07TR | HER07A07_M13R |
| LQCBT08TFB | HER08A08_M13F | LQCBT08TR | HER08A08_M13R |
| LQCBT09TFB | HER09A09_M13F | LQCBT09TR | HER09A09_M13R |
| LQCBT10TFB | HER10A10_M13F | LQCBT10TR | HER10A10_M13R |
| LQCBT11TFB | HER11A11_M13F | LQCBT11TR | HER11A11_M13R |
| LQCBT12TFB | HER12A12_M13F | LQCBT12TR | HER12A12_M13R |
| LQCBT13TFB | HER01B01_M13F | LQCBT13TR | HER01B01_M13R |
| LQCBT14TFB | HER02B02_M13F | LQCBT14TR | HER02B02_M13R |
| LQCBT15TFB | HER03B03_M13F | LQCBT15TR | HER03B03_M13R |
| LQCBT16TFB | HER04B04_M13F | LQCBT16TR | HER04B04_M13R |
| LQCBT17TFB | HER05B05_M13F | LQCBT17TR | HER05B05_M13R |
| LQCBT18TFB | HER06B06_M13F | LQCBT18TR | HER06B06_M13R |
| LQCBT19TFB | HER07B07_M13F | LQCBT19TR | HER07B07_M13R |
| LQCBT20TFB | HER08B08_M13F | LQCBT20TR | HER08B08_M13R |
| LQCBT21TFB | HER09B09_M13F | LQCBT21TR | HER09B09_M13R |
| LQCBT22TFB | HER10B10_M13F | LQCBT22TR | HER10B10_M13R |
| LQCBT23TFB | HER11B11_M13F | LQCBT23TR | HER11B11_M13R |
| LQCBT24TFB | HER12B12_M13F | LQCBT24TR | HER12B12_M13R |
| LQCBT25TFB | HER01C01_M13F | LQCBT25TR | HER01C01_M13R |
| LQCBT26TFB | HER02C02_M13F | LQCBT26TR | HER02C02_M13R |
| LQCBT27TFB | HER03C03_M13F | LQCBT27TR | HER03C03_M13R |
| LQCBT28TFB | HER04C04_M13F | LQCBT28TR | HER04C04_M13R |
| LQCBT29TFB | HER05C05_M13F | LQCBT29TR | HER05C05_M13R |
| LQCBT30TFB | HER06C06_M13F | LQCBT30TR | HER06C06_M13R |
| LQCBT31TFB | HER07C07_M13F | LQCBT31TR | HER07C07_M13R |
| LQCBT32TFB | HER08C08_M13F | LQCBT32TR | HER08C08_M13R |
| LQCBT33TFB | HER09C09_M13F | LQCBT33TR | HER09C09_M13R |
| LQCBT34TFB | HER10C10_M13F | LQCBT34TR | HER10C10_M13R |
| LQCBT35TFB | HER11C11_M13F | LQCBT35TR | HER11C11_M13R |
| LQCBT36TFB | HER12C12_M13F | LQCBT36TR | HER12C12_M13R |
| LQCBT37TFB | HER01D01_M13F | LQCBT37TR | HER01D01_M13R |
| LQCBT38TFB | HER02D02_M13F | LQCBT38TR | HER02D02_M13R |
| LQCBT39TFB | HER03D03_M13F | LQCBT39TR | HER03D03_M13R |
| LQCBT40TFB | HER04D04_M13F | LQCBT40TR | HER04D04_M13R |
| LQCBT41TFB | HER05D05_M13F | LQCBT41TR | HER05D05_M13R |
| LQCBT42TFB | HER06D06_M13F | LQCBT42TR | HER06D06_M13R |
| LQCBT43TFB | HER07D07_M13F | LQCBT43TR | HER07D07_M13R |
| LQCBT44TFB | HER08D08_M13F | LQCBT44TR | HER08D08_M13R |
| LQCBT45TFB | HER09D09_M13F | LQCBT45TR | HER09D09_M13R |
| LQCBT46TFB | HER10D10_M13F | LQCBT46TR | HER10D10_M13R |
| LQCBT47TFB | HER11D11_M13F | LQCBT47TR | HER11D11_M13R |
| LQCBT48TFB | HER12D12_M13F | LQCBT48TR | HER12D12_M13R |
| LQCBT49TFB | HER01E01_M13F | LQCBT49TR | HER01E01_M13R |
| LQCBT50TFB | HER02E02_M13F | LQCBT50TR | HER02E02_M13R |
| LQCBT51TFB | HER03E03_M13F | LQCBT51TR | HER03E03_M13R |
| LQCBT52TFB | HER04E04_M13F | LQCBT52TR | HER04E04_M13R |
| LQCBT53TFB | HER05E05_M13F | LQCBT53TR | HER05E05_M13R |
| LQCBT54TFB | HER06E06_M13F | LQCBT54TR | HER06E06_M13R |
| LQCBT55TFB | HER07E07_M13F | LQCBT55TR | HER07E07_M13R |
| LQCBT56TFB | HER08E08_M13F | LQCBT56TR | HER08E08_M13R |
| LQCBT57TFB | HER09E09_M13F | LQCBT57TR | HER09E09_M13R |
| LQCBT58TFB | HER10E10_M13F | LQCBT58TR | HER10E10_M13R |
| LQCBT59TFB | HER11E11_M13F | LQCBT59TR | HER11E11_M13R |
| LQCBT60TFB | HER12E12_M13F | LQCBT60TR | HER12E12_M13R |
| LQCBT61TFB | HER01F01_M13F | LQCBT61TR | HER01F01_M13R |
| LQCBT62TFB | HER02F02_M13F | LQCBT62TR | HER02F02_M13R |
| LQCBT63TFB | HER03F03_M13F | LQCBT63TR | HER03F03_M13R |
| LQCBT64TFB | HER04F04_M13F | LQCBT64TR | HER04F04_M13R |
| LQCBT65TFB | HER05F05_M13F | LQCBT65TR | HER05F05_M13R |
| LQCBT66TFB | HER06F06_M13F | LQCBT66TR | HER06F06_M13R |
| LQCBT67TFB | HER07F07_M13F | LQCBT67TR | HER07F07_M13R |
| LQCBT68TFB | HER08F08_M13F | LQCBT68TR | HER08F08_M13R |
| LQCBT69TFB | HER09F09_M13F | LQCBT69TR | HER09F09_M13R |
| LQCBT70TFB | HER10F10_M13F | LQCBT70TR | HER10F10_M13R |
| LQCBT71TFB | HER11F11_M13F | LQCBT71TR | HER11F11_M13R |
| LQCBT72TFB | HER12F12_M13F | LQCBT72TR | HER12F12_M13R |
| LQCBT73TFB | HER01G01_M13F | LQCBT73TR | HER01G01_M13R |
| LQCBT74TFB | HER02G02_M13F | LQCBT74TR | HER02G02_M13R |
| LQCBT75TFB | HER03G03_M13F | LQCBT75TR | HER03G03_M13R |
| LQCBT76TFB | HER04G04_M13F | LQCBT76TR | HER04G04_M13R |
| LQCBT77TFB | HER05G05_M13F | LQCBT77TR | HER05G05_M13R |
| LQCBT78TFB | HER06G06_M13F | LQCBT78TR | HER06G06_M13R |
| LQCBT79TFB | HER07G07_M13F | LQCBT79TR | HER07G07_M13R |
| LQCBT80TFB | HER08G08_M13F | LQCBT80TR | HER08G08_M13R |
| LQCBT81TFB | HER09G09_M13F | LQCBT81TR | HER09G09_M13R |
| LQCBT82TFB | HER10G10_M13F | LQCBT82TR | HER10G10_M13R |
| LQCBT83TFB | HER11G11_M13F | LQCBT83TR | HER11G11_M13R |
| LQCBT84TFB | HER12G12_M13F | LQCBT84TR | HER12G12_M13R |
| LQCBT85TFB | HER01H01_M13F | LQCBT85TR | HER01H01_M13R |
| LQCBT86TFB | HER02H02_M13F | LQCBT86TR | HER02H02_M13R |
| LQCBT87TFB | HER03H03_M13F | LQCBT87TR | HER03H03_M13R |
| LQCBT88TFB | HER04H04_M13F | LQCBT88TR | HER04H04_M13R |
| LQCBT89TFB | HER05H05_M13F | LQCBT89TR | HER05H05_M13R |
| LQCBT90TFB | HER06H06_M13F | LQCBT90TR | HER06H06_M13R |
| LQCBT91TFB | HER07H07_M13F | LQCBT91TR | HER07H07_M13R |
| LQCBT92TFB | HER08H08_M13F | LQCBT92TR | HER08H08_M13R |
| LQCBT93TFB | HER09H09_M13F | LQCBT93TR | HER09H09_M13R |
| LQCBT94TFB | HER10H10_M13F | LQCBT94TR | HER10H10_M13R |
| LQCBT95TFB | HER11H11_M13F | LQCBT95TR | HER11H11_M13R |
| LQCBT96TFB | HER12H12_M13F | LQCBT96TR | HER12H12_M13R |

Table S1-C. Correspondence of *Heliothis virescens Bam*HI BAC end sequence names with their library clone names

| TIGR name | Library clone name_primer | TIGR name | Library clone name_primer |
| --- | --- | --- | --- |
| LQCBW01TFB | HVB01A01_M13F | LQCBW01TR | HVB01A01_M13R |
| LQCBW02TFB | HVB02A02_M13F | LQCBW02TR | HVB02A02_M13R |
| LQCBW03TFB | HVB03A03_M13F | LQCBW03TR | HVB03A03_M13R |
| LQCBW04TFB | HVB04A04_M13F | LQCBW04TR | HVB04A04_M13R |
| LQCBW05TFB | HVB05A05_M13F | LQCBW05TR | HVB05A05_M13R |
| LQCBW06TFB | HVB06A06_M13F | LQCBW06TR | HVB06A06_M13R |
| LQCBW07TFB | HVB07A07_M13F | LQCBW07TR | HVB07A07_M13R |
| LQCBW08TFB | HVB08A08_M13F | LQCBW08TR | HVB08A08_M13R |
| LQCBW09TFB | HVB09A09_M13F | LQCBW09TR | HVB09A09_M13R |
| LQCBW10TFB | HVB10A10_M13F | LQCBW10TR | HVB10A10_M13R |
| LQCBW11TFB | HVB11A11_M13F | LQCBW11TR | HVB11A11_M13R |
| LQCBW12TFB | HVB12A12_M13F | LQCBW12TR | HVB12A12_M13R |
| LQCBW13TFB | HVB01B01_M13F | LQCBW13TR | HVB01B01_M13R |
| LQCBW14TFB | HVB02B02_M13F | LQCBW14TR | HVB02B02_M13R |
| LQCBW15TFB | HVB03B03_M13F | LQCBW15TR | HVB03B03_M13R |
| LQCBW16TFB | HVB04B04_M13F | LQCBW16TR | HVB04B04_M13R |
| LQCBW17TFB | HVB05B05_M13F | LQCBW17TR | HVB05B05_M13R |
| LQCBW18TFB | HVB06B06_M13F | LQCBW18TR | HVB06B06_M13R |
| LQCBW19TFB | HVB07B07_M13F | LQCBW19TR | HVB07B07_M13R |
| LQCBW20TFB | HVB08B08_M13F | LQCBW20TR | HVB08B08_M13R |
| LQCBW21TFB | HVB09B09_M13F | LQCBW21TR | HVB09B09_M13R |
| LQCBW22TFB | HVB10B10_M13F | LQCBW22TR | HVB10B10_M13R |
| LQCBW23TFB | HVB11B11_M13F | LQCBW23TR | HVB11B11_M13R |
| LQCBW24TFB | HVB12B12_M13F | LQCBW24TR | HVB12B12_M13R |
| LQCBW25TFB | HVB01C01_M13F | LQCBW25TR | HVB01C01_M13R |
| LQCBW26TFB | HVB02C02_M13F | LQCBW26TR | HVB02C02_M13R |
| LQCBW27TFB | HVB03C03_M13F | LQCBW27TR | HVB03C03_M13R |
| LQCBW28TFB | HVB04C04_M13F | LQCBW28TR | HVB04C04_M13R |
| LQCBW29TFB | HVB05C05_M13F | LQCBW29TR | HVB05C05_M13R |
| LQCBW30TFB | HVB06C06_M13F | LQCBW30TR | HVB06C06_M13R |
| LQCBW31TFB | HVB07C07_M13F | LQCBW31TR | HVB07C07_M13R |
| LQCBW32TFB | HVB08C08_M13F | LQCBW32TR | HVB08C08_M13R |
| LQCBW33TFB | HVB09C09_M13F | LQCBW33TR | HVB09C09_M13R |
| LQCBW34TFB | HVB10C10_M13F | LQCBW34TR | HVB10C10_M13R |
| LQCBW35TFB | HVB11C11_M13F | LQCBW35TR | HVB11C11_M13R |
| LQCBW36TFB | HVB12C12_M13F | LQCBW36TR | HVB12C12_M13R |
| LQCBW37TFB | HVB01D01_M13F | LQCBW37TR | HVB01D01_M13R |
| LQCBW38TFB | HVB02D02_M13F | LQCBW38TR | HVB02D02_M13R |
| LQCBW39TFB | HVB03D03_M13F | LQCBW39TR | HVB03D03_M13R |
| LQCBW40TFB | HVB04D04_M13F | LQCBW40TR | HVB04D04_M13R |
| LQCBW41TFB | HVB05D05_M13F | LQCBW41TR | HVB05D05_M13R |
| LQCBW42TFB | HVB06D06_M13F | LQCBW42TR | HVB06D06_M13R |
| LQCBW43TFB | HVB07D07_M13F | LQCBW43TR | HVB07D07_M13R |
| LQCBW44TFB | HVB08D08_M13F | LQCBW44TR | HVB08D08_M13R |
| LQCBW45TFB | HVB09D09_M13F | LQCBW45TR | HVB09D09_M13R |
| LQCBW46TFB | HVB10D10_M13F | LQCBW46TR | HVB10D10_M13R |
| LQCBW47TFB | HVB11D11_M13F | LQCBW47TR | HVB11D11_M13R |
| LQCBW48TFB | HVB12D12_M13F | LQCBW48TR | HVB12D12_M13R |
| LQCBW49TFB | HVB01E01_M13F | LQCBW49TR | HVB01E01_M13R |
| LQCBW50TFB | HVB02E02_M13F | LQCBW50TR | HVB02E02_M13R |
| LQCBW51TFB | HVB03E03_M13F | LQCBW51TR | HVB03E03_M13R |
| LQCBW52TFB | HVB04E04_M13F | LQCBW52TR | HVB04E04_M13R |
| LQCBW53TFB | HVB05E05_M13F | LQCBW53TR | HVB05E05_M13R |
| LQCBW54TFB | HVB06E06_M13F | LQCBW54TR | HVB06E06_M13R |
| LQCBW55TFB | HVB07E07_M13F | LQCBW55TR | HVB07E07_M13R |
| LQCBW56TFB | HVB08E08_M13F | LQCBW56TR | HVB08E08_M13R |
| LQCBW57TFB | HVB09E09_M13F | LQCBW57TR | HVB09E09_M13R |
| LQCBW58TFB | HVB10E10_M13F | LQCBW58TR | HVB10E10_M13R |
| LQCBW59TFB | HVB11E11_M13F | LQCBW59TR | HVB11E11_M13R |
| LQCBW60TFB | HVB12E12_M13F | LQCBW60TR | HVB12E12_M13R |
| LQCBW61TFB | HVB01F01_M13F | LQCBW61TR | HVB01F01_M13R |
| LQCBW62TFB | HVB02F02_M13F | LQCBW62TR | HVB02F02_M13R |
| LQCBW63TFB | HVB03F03_M13F | LQCBW63TR | HVB03F03_M13R |
| LQCBW64TFB | HVB04F04_M13F | LQCBW64TR | HVB04F04_M13R |
| LQCBW65TFB | HVB05F05_M13F | LQCBW65TR | HVB05F05_M13R |
| LQCBW66TFB | HVB06F06_M13F | LQCBW66TR | HVB06F06_M13R |
| LQCBW67TFB | HVB07F07_M13F | LQCBW67TR | HVB07F07_M13R |
| LQCBW68TFB | HVB08F08_M13F | LQCBW68TR | HVB08F08_M13R |
| LQCBW69TFB | HVB09F09_M13F | LQCBW69TR | HVB09F09_M13R |
| LQCBW70TFB | HVB10F10_M13F | LQCBW70TR | HVB10F10_M13R |
| LQCBW71TFB | HVB11F11_M13F | LQCBW71TR | HVB11F11_M13R |
| LQCBW72TFB | HVB12F12_M13F | LQCBW72TR | HVB12F12_M13R |
| LQCBW73TFB | HVB01G01_M13F | LQCBW73TR | HVB01G01_M13R |
| LQCBW74TFB | HVB02G02_M13F | LQCBW74TR | HVB02G02_M13R |
| LQCBW75TFB | HVB03G03_M13F | LQCBW75TR | HVB03G03_M13R |
| LQCBW76TFB | HVB04G04_M13F | LQCBW76TR | HVB04G04_M13R |
| LQCBW77TFB | HVB05G05_M13F | LQCBW77TR | HVB05G05_M13R |
| LQCBW78TFB | HVB06G06_M13F | LQCBW78TR | HVB06G06_M13R |
| LQCBW79TFB | HVB07G07_M13F | LQCBW79TR | HVB07G07_M13R |
| LQCBW80TFB | HVB08G08_M13F | LQCBW80TR | HVB08G08_M13R |
| LQCBW81TFB | HVB09G09_M13F | LQCBW81TR | HVB09G09_M13R |
| LQCBW82TFB | HVB10G10_M13F | LQCBW82TR | HVB10G10_M13R |
| LQCBW83TFB | HVB11G11_M13F | LQCBW83TR | HVB11G11_M13R |
| LQCBW84TFB | HVB12G12_M13F | LQCBW84TR | HVB12G12_M13R |
| LQCBW85TFB | HVB01H01_M13F | LQCBW85TR | HVB01H01_M13R |
| LQCBW86TFB | HVB02H02_M13F | LQCBW86TR | HVB02H02_M13R |
| LQCBW87TFB | HVB03H03_M13F | LQCBW87TR | HVB03H03_M13R |
| LQCBW88TFB | HVB04H04_M13F | LQCBW88TR | HVB04H04_M13R |
| LQCBW89TFB | HVB05H05_M13F | LQCBW89TR | HVB05H05_M13R |
| LQCBW90TFB | HVB06H06_M13F | LQCBW90TR | HVB06H06_M13R |
| LQCBW91TFB | HVB07H07_M13F | LQCBW91TR | HVB07H07_M13R |
| LQCBW92TFB | HVB08H08_M13F | LQCBW92TR | HVB08H08_M13R |
| LQCBW93TFB | HVB09H09_M13F | LQCBW93TR | HVB09H09_M13R |
| LQCBW94TFB | HVB10H10_M13F | LQCBW94TR | HVB10H10_M13R |
| LQCBW95TFB | HVB11H11_M13F | LQCBW95TR | HVB11H11_M13R |
| LQCBW96TFB | HVB12H12_M13F | LQCBW96TR | HVB12H12_M13R |

Table S1-D. Correspondence of *Heliothis virescens Eco*RI BAC end sequence names with their library clone names

| TIGR name | Library clone name_primer | TIGR name | Library clone name_primer |
| --- | --- | --- | --- |
| LQCBV01TFB | HVR01A01_M13F | LQCBV01TR | HVR01A01_M13R |
| LQCBV02TFB | HVR02A02_M13F | LQCBV02TR | HVR02A02_M13R |
| LQCBV03TFB | HVR03A03_M13F | LQCBV03TR | HVR03A03_M13R |
| LQCBV04TFB | HVR04A04_M13F | LQCBV04TR | HVR04A04_M13R |
| LQCBV05TFB | HVR05A05_M13F | LQCBV05TR | HVR05A05_M13R |
| LQCBV06TFB | HVR06A06_M13F | LQCBV06TR | HVR06A06_M13R |
| LQCBV07TFB | HVR07A07_M13F | LQCBV07TR | HVR07A07_M13R |
| LQCBV08TFB | HVR08A08_M13F | LQCBV08TR | HVR08A08_M13R |
| LQCBV09TFB | HVR09A09_M13F | LQCBV09TR | HVR09A09_M13R |
| LQCBV10TFB | HVR10A10_M13F | LQCBV10TR | HVR10A10_M13R |
| LQCBV11TFB | HVR11A11_M13F | LQCBV11TR | HVR11A11_M13R |
| LQCBV12TFB | HVR12A12_M13F | LQCBV12TR | HVR12A12_M13R |
| LQCBV13TFB | HVR01B01_M13F | LQCBV13TR | HVR01B01_M13R |
| LQCBV14TFB | HVR02B02_M13F | LQCBV14TR | HVR02B02_M13R |
| LQCBV15TFB | HVR03B03_M13F | LQCBV15TR | HVR03B03_M13R |
| LQCBV16TFB | HVR04B04_M13F | LQCBV16TR | HVR04B04_M13R |
| LQCBV17TFB | HVR05B05_M13F | LQCBV17TR | HVR05B05_M13R |
| LQCBV18TFB | HVR06B06_M13F | LQCBV18TR | HVR06B06_M13R |
| LQCBV19TFB | HVR07B07_M13F | LQCBV19TR | HVR07B07_M13R |
| LQCBV20TFB | HVR08B08_M13F | LQCBV20TR | HVR08B08_M13R |
| LQCBV21TFB | HVR09B09_M13F | LQCBV21TR | HVR09B09_M13R |
| LQCBV22TFB | HVR10B10_M13F | LQCBV22TR | HVR10B10_M13R |
| LQCBV23TFB | HVR11B11_M13F | LQCBV23TR | HVR11B11_M13R |
| LQCBV24TFB | HVR12B12_M13F | LQCBV24TR | HVR12B12_M13R |
| LQCBV25TFB | HVR01C01_M13F | LQCBV25TR | HVR01C01_M13R |
| LQCBV26TFB | HVR02C02_M13F | LQCBV26TR | HVR02C02_M13R |
| LQCBV27TFB | HVR03C03_M13F | LQCBV27TR | HVR03C03_M13R |
| LQCBV28TFB | HVR04C04_M13F | LQCBV28TR | HVR04C04_M13R |
| LQCBV29TFB | HVR05C05_M13F | LQCBV29TR | HVR05C05_M13R |
| LQCBV30TFB | HVR06C06_M13F | LQCBV30TR | HVR06C06_M13R |
| LQCBV31TFB | HVR07C07_M13F | LQCBV31TR | HVR07C07_M13R |
| LQCBV32TFB | HVR08C08_M13F | LQCBV32TR | HVR08C08_M13R |
| LQCBV33TFB | HVR09C09_M13F | LQCBV33TR | HVR09C09_M13R |
| LQCBV34TFB | HVR10C10_M13F | LQCBV34TR | HVR10C10_M13R |
| LQCBV35TFB | HVR11C11_M13F | LQCBV35TR | HVR11C11_M13R |
| LQCBV36TFB | HVR12C12_M13F | LQCBV36TR | HVR12C12_M13R |
| LQCBV37TFB | HVR01D01_M13F | LQCBV37TR | HVR01D01_M13R |
| LQCBV38TFB | HVR02D02_M13F | LQCBV38TR | HVR02D02_M13R |
| LQCBV39TFB | HVR03D03_M13F | LQCBV39TR | HVR03D03_M13R |
| LQCBV40TFB | HVR04D04_M13F | LQCBV40TR | HVR04D04_M13R |
| LQCBV41TFB | HVR05D05_M13F | LQCBV41TR | HVR05D05_M13R |
| LQCBV42TFB | HVR06D06_M13F | LQCBV42TR | HVR06D06_M13R |
| LQCBV43TFB | HVR07D07_M13F | LQCBV43TR | HVR07D07_M13R |
| LQCBV44TFB | HVR08D08_M13F | LQCBV44TR | HVR08D08_M13R |
| LQCBV45TFB | HVR09D09_M13F | LQCBV45TR | HVR09D09_M13R |
| LQCBV46TFB | HVR10D10_M13F | LQCBV46TR | HVR10D10_M13R |
| LQCBV47TFB | HVR11D11_M13F | LQCBV47TR | HVR11D11_M13R |
| LQCBV48TFB | HVR12D12_M13F | LQCBV48TR | HVR12D12_M13R |
| LQCBV49TFB | HVR01E01_M13F | LQCBV49TR | HVR01E01_M13R |
| LQCBV50TFB | HVR02E02_M13F | LQCBV50TR | HVR02E02_M13R |
| LQCBV51TFB | HVR03E03_M13F | LQCBV51TR | HVR03E03_M13R |
| LQCBV52TFB | HVR04E04_M13F | LQCBV52TR | HVR04E04_M13R |
| LQCBV53TFB | HVR05E05_M13F | LQCBV53TR | HVR05E05_M13R |
| LQCBV54TFB | HVR06E06_M13F | LQCBV54TR | HVR06E06_M13R |
| LQCBV55TFB | HVR07E07_M13F | LQCBV55TR | HVR07E07_M13R |
| LQCBV56TFB | HVR08E08_M13F | LQCBV56TR | HVR08E08_M13R |
| LQCBV57TFB | HVR09E09_M13F | LQCBV57TR | HVR09E09_M13R |
| LQCBV58TFB | HVR10E10_M13F | LQCBV58TR | HVR10E10_M13R |
| LQCBV59TFB | HVR11E11_M13F | LQCBV59TR | HVR11E11_M13R |
| LQCBV60TFB | HVR12E12_M13F | LQCBV60TR | HVR12E12_M13R |
| LQCBV61TFB | HVR01F01_M13F | LQCBV61TR | HVR01F01_M13R |
| LQCBV62TFB | HVR02F02_M13F | LQCBV62TR | HVR02F02_M13R |
| LQCBV63TFB | HVR03F03_M13F | LQCBV63TR | HVR03F03_M13R |
| LQCBV64TFB | HVR04F04_M13F | LQCBV64TR | HVR04F04_M13R |
| LQCBV65TFB | HVR05F05_M13F | LQCBV65TR | HVR05F05_M13R |
| LQCBV66TFB | HVR06F06_M13F | LQCBV66TR | HVR06F06_M13R |
| LQCBV67TFB | HVR07F07_M13F | LQCBV67TR | HVR07F07_M13R |
| LQCBV68TFB | HVR08F08_M13F | LQCBV68TR | HVR08F08_M13R |
| LQCBV69TFB | HVR09F09_M13F | LQCBV69TR | HVR09F09_M13R |
| LQCBV70TFB | HVR10F10_M13F | LQCBV70TR | HVR10F10_M13R |
| LQCBV71TFB | HVR11F11_M13F | LQCBV71TR | HVR11F11_M13R |
| LQCBV72TFB | HVR12F12_M13F | LQCBV72TR | HVR12F12_M13R |
| LQCBV73TFB | HVR01G01_M13F | LQCBV73TR | HVR01G01_M13R |
| LQCBV74TFB | HVR02G02_M13F | LQCBV74TR | HVR02G02_M13R |
| LQCBV75TFB | HVR03G03_M13F | LQCBV75TR | HVR03G03_M13R |
| LQCBV76TFB | HVR04G04_M13F | LQCBV76TR | HVR04G04_M13R |
| LQCBV77TFB | HVR05G05_M13F | LQCBV77TR | HVR05G05_M13R |
| LQCBV78TFB | HVR06G06_M13F | LQCBV78TR | HVR06G06_M13R |
| LQCBV79TFB | HVR07G07_M13F | LQCBV79TR | HVR07G07_M13R |
| LQCBV80TFB | HVR08G08_M13F | LQCBV80TR | HVR08G08_M13R |
| LQCBV81TFB | HVR09G09_M13F | LQCBV81TR | HVR09G09_M13R |
| LQCBV82TFB | HVR10G10_M13F | LQCBV82TR | HVR10G10_M13R |
| LQCBV83TFB | HVR11G11_M13F | LQCBV83TR | HVR11G11_M13R |
| LQCBV84TFB | HVR12G12_M13F | LQCBV84TR | HVR12G12_M13R |
| LQCBV85TFB | HVR01H01_M13F | LQCBV85TR | HVR01H01_M13R |
| LQCBV86TFB | HVR02H02_M13F | LQCBV86TR | HVR02H02_M13R |
| LQCBV87TFB | HVR03H03_M13F | LQCBV87TR | HVR03H03_M13R |
| LQCBV88TFB | HVR04H04_M13F | LQCBV88TR | HVR04H04_M13R |
| LQCBV89TFB | HVR05H05_M13F | LQCBV89TR | HVR05H05_M13R |
| LQCBV90TFB | HVR06H06_M13F | LQCBV90TR | HVR06H06_M13R |
| LQCBV91TFB | HVR07H07_M13F | LQCBV91TR | HVR07H07_M13R |
| LQCBV92TFB | HVR08H08_M13F | LQCBV92TR | HVR08H08_M13R |
| LQCBV93TFB | HVR09H09_M13F | LQCBV93TR | HVR09H09_M13R |
| LQCBV94TFB | HVR10H10_M13F | LQCBV94TR | HVR10H10_M13R |
| LQCBV95TFB | HVR11H11_M13F | LQCBV95TR | HVR11H11_M13R |
| LQCBV96TFB | HVR12H12_M13F | LQCBV96TR | HVR12H12_M13R |

Table S1-E. Correspondence of *Manduca sexta Bam*HI BAC end sequence nameswith their library clone names

| TIGR name | Library clone name_primer | TIGR name | Library clone name_primer |
| --- | --- | --- | --- |
| LQCBY01TFB | MSB01A01_M13F | LQCBY01TR | MSB01A01_M13R |
| LQCBY02TFB | MSB02A02_M13F | LQCBY02TR | MSB02A02_M13R |
| LQCBY03TFB | MSB03A03_M13F | LQCBY03TR | MSB03A03_M13R |
| LQCBY04TFB | MSB04A04_M13F | LQCBY04TR | MSB04A04_M13R |
| LQCBY05TFB | MSB05A05_M13F | LQCBY05TR | MSB05A05_M13R |
| LQCBY06TFB | MSB06A06_M13F | LQCBY06TR | MSB06A06_M13R |
| LQCBY07TFB | MSB07A07_M13F | LQCBY07TR | MSB07A07_M13R |
| LQCBY08TFB | MSB08A08_M13F | LQCBY08TR | MSB08A08_M13R |
| LQCBY09TFB | MSB09A09_M13F | LQCBY09TR | MSB09A09_M13R |
| LQCBY10TFB | MSB10A10_M13F | LQCBY10TR | MSB10A10_M13R |
| LQCBY11TFB | MSB11A11_M13F | LQCBY11TR | MSB11A11_M13R |
| LQCBY12TFB | MSB12A12_M13F | LQCBY12TR | MSB12A12_M13R |
| LQCBY13TFB | MSB01B01_M13F | LQCBY13TR | MSB01B01_M13R |
| LQCBY14TFB | MSB02B02_M13F | LQCBY14TR | MSB02B02_M13R |
| LQCBY15TFB | MSB03B03_M13F | LQCBY15TR | MSB03B03_M13R |
| LQCBY16TFB | MSB04B04_M13F | LQCBY16TR | MSB04B04_M13R |
| LQCBY17TFB | MSB05B05_M13F | LQCBY17TR | MSB05B05_M13R |
| LQCBY18TFB | MSB06B06_M13F | LQCBY18TR | MSB06B06_M13R |
| LQCBY19TFB | MSB07B07_M13F | LQCBY19TR | MSB07B07_M13R |
| LQCBY20TFB | MSB08B08_M13F | LQCBY20TR | MSB08B08_M13R |
| LQCBY21TFB | MSB09B09_M13F | LQCBY21TR | MSB09B09_M13R |
| LQCBY22TFB | MSB10B10_M13F | LQCBY22TR | MSB10B10_M13R |
| LQCBY23TFB | MSB11B11_M13F | LQCBY23TR | MSB11B11_M13R |
| LQCBY24TFB | MSB12B12_M13F | LQCBY24TR | MSB12B12_M13R |
| LQCBY25TFB | MSB01C01_M13F | LQCBY25TR | MSB01C01_M13R |
| LQCBY26TFB | MSB02C02_M13F | LQCBY26TR | MSB02C02_M13R |
| LQCBY27TFB | MSB03C03_M13F | LQCBY27TR | MSB03C03_M13R |
| LQCBY28TFB | MSB04C04_M13F | LQCBY28TR | MSB04C04_M13R |
| LQCBY29TFB | MSB05C05_M13F | LQCBY29TR | MSB05C05_M13R |
| LQCBY30TFB | MSB06C06_M13F | LQCBY30TR | MSB06C06_M13R |
| LQCBY31TFB | MSB07C07_M13F | LQCBY31TR | MSB07C07_M13R |
| LQCBY32TFB | MSB08C08_M13F | LQCBY32TR | MSB08C08_M13R |
| LQCBY33TFB | MSB09C09_M13F | LQCBY33TR | MSB09C09_M13R |
| LQCBY34TFB | MSB10C10_M13F | LQCBY34TR | MSB10C10_M13R |
| LQCBY35TFB | MSB11C11_M13F | LQCBY35TR | MSB11C11_M13R |
| LQCBY36TFB | MSB12C12_M13F | LQCBY36TR | MSB12C12_M13R |
| LQCBY37TFB | MSB01D01_M13F | LQCBY37TR | MSB01D01_M13R |
| LQCBY38TFB | MSB02D02_M13F | LQCBY38TR | MSB02D02_M13R |
| LQCBY39TFB | MSB03D03_M13F | LQCBY39TR | MSB03D03_M13R |
| LQCBY40TFB | MSB04D04_M13F | LQCBY40TR | MSB04D04_M13R |
| LQCBY41TFB | MSB05D05_M13F | LQCBY41TR | MSB05D05_M13R |
| LQCBY42TFB | MSB06D06_M13F | LQCBY42TR | MSB06D06_M13R |
| LQCBY43TFB | MSB07D07_M13F | LQCBY43TR | MSB07D07_M13R |
| LQCBY44TFB | MSB08D08_M13F | LQCBY44TR | MSB08D08_M13R |
| LQCBY45TFB | MSB09D09_M13F | LQCBY45TR | MSB09D09_M13R |
| LQCBY46TFB | MSB10D10_M13F | LQCBY46TR | MSB10D10_M13R |
| LQCBY47TFB | MSB11D11_M13F | LQCBY47TR | MSB11D11_M13R |
| LQCBY48TFB | MSB12D12_M13F | LQCBY48TR | MSB12D12_M13R |
| LQCBY49TFB | MSB01E01_M13F | LQCBY49TR | MSB01E01_M13R |
| LQCBY50TFB | MSB02E02_M13F | LQCBY50TR | MSB02E02_M13R |
| LQCBY51TFB | MSB03E03_M13F | LQCBY51TR | MSB03E03_M13R |
| LQCBY52TFB | MSB04E04_M13F | LQCBY52TR | MSB04E04_M13R |
| LQCBY53TFB | MSB05E05_M13F | LQCBY53TR | MSB05E05_M13R |
| LQCBY54TFB | MSB06E06_M13F | LQCBY54TR | MSB06E06_M13R |
| LQCBY55TFB | MSB07E07_M13F | LQCBY55TR | MSB07E07_M13R |
| LQCBY56TFB | MSB08E08_M13F | LQCBY56TR | MSB08E08_M13R |
| LQCBY57TFB | MSB09E09_M13F | LQCBY57TR | MSB09E09_M13R |
| LQCBY58TFB | MSB10E10_M13F | LQCBY58TR | MSB10E10_M13R |
| LQCBY59TFB | MSB11E11_M13F | LQCBY59TR | MSB11E11_M13R |
| LQCBY60TFB | MSB12E12_M13F | LQCBY60TR | MSB12E12_M13R |
| LQCBY61TFB | MSB01F01_M13F | LQCBY61TR | MSB01F01_M13R |
| LQCBY62TFB | MSB02F02_M13F | LQCBY62TR | MSB02F02_M13R |
| LQCBY63TFB | MSB03F03_M13F | LQCBY63TR | MSB03F03_M13R |
| LQCBY64TFB | MSB04F04_M13F | LQCBY64TR | MSB04F04_M13R |
| LQCBY65TFB | MSB05F05_M13F | LQCBY65TR | MSB05F05_M13R |
| LQCBY66TFB | MSB06F06_M13F | LQCBY66TR | MSB06F06_M13R |
| LQCBY67TFB | MSB07F07_M13F | LQCBY67TR | MSB07F07_M13R |
| LQCBY68TFB | MSB08F08_M13F | LQCBY68TR | MSB08F08_M13R |
| LQCBY69TFB | MSB09F09_M13F | LQCBY69TR | MSB09F09_M13R |
| LQCBY70TFB | MSB10F10_M13F | LQCBY70TR | MSB10F10_M13R |
| LQCBY71TFB | MSB11F11_M13F | LQCBY71TR | MSB11F11_M13R |
| LQCBY72TFB | MSB12F12_M13F | LQCBY72TR | MSB12F12_M13R |
| LQCBY73TFB | MSB01G01_M13F | LQCBY73TR | MSB01G01_M13R |
| LQCBY74TFB | MSB02G02_M13F | LQCBY74TR | MSB02G02_M13R |
| LQCBY75TFB | MSB03G03_M13F | LQCBY75TR | MSB03G03_M13R |
| LQCBY76TFB | MSB04G04_M13F | LQCBY76TR | MSB04G04_M13R |
| LQCBY77TFB | MSB05G05_M13F | LQCBY77TR | MSB05G05_M13R |
| LQCBY78TFB | MSB06G06_M13F | LQCBY78TR | MSB06G06_M13R |
| LQCBY79TFB | MSB07G07_M13F | LQCBY79TR | MSB07G07_M13R |
| LQCBY80TFB | MSB08G08_M13F | LQCBY80TR | MSB08G08_M13R |
| LQCBY81TFB | MSB09G09_M13F | LQCBY81TR | MSB09G09_M13R |
| LQCBY82TFB | MSB10G10_M13F | LQCBY82TR | MSB10G10_M13R |
| LQCBY83TFB | MSB11G11_M13F | LQCBY83TR | MSB11G11_M13R |
| LQCBY84TFB | MSB12G12_M13F | LQCBY84TR | MSB12G12_M13R |
| LQCBY85TFB | MSB01H01_M13F | LQCBY85TR | MSB01H01_M13R |
| LQCBY86TFB | MSB02H02_M13F | LQCBY86TR | MSB02H02_M13R |
| LQCBY87TFB | MSB03H03_M13F | LQCBY87TR | MSB03H03_M13R |
| LQCBY88TFB | MSB04H04_M13F | LQCBY88TR | MSB04H04_M13R |
| LQCBY89TFB | MSB05H05_M13F | LQCBY89TR | MSB05H05_M13R |
| LQCBY90TFB | MSB06H06_M13F | LQCBY90TR | MSB06H06_M13R |
| LQCBY91TFB | MSB07H07_M13F | LQCBY91TR | MSB07H07_M13R |
| LQCBY92TFB | MSB08H08_M13F | LQCBY92TR | MSB08H08_M13R |
| LQCBY93TFB | MSB09H09_M13F | LQCBY93TR | MSB09H09_M13R |
| LQCBY94TFB | MSB10H10_M13F | LQCBY94TR | MSB10H10_M13R |
| LQCBY95TFB | MSB11H11_M13F | LQCBY95TR | MSB11H11_M13R |
| LQCBY96TFB | MSB12H12_M13F | LQCBY96TR | MSB12H12_M13R |

Table S1-F. Correspondence of *Manduca sexta Eco*RI BAC end sequence names with their library clone names

| TIGR name | Library clone name_primer | TIGR name | Library clone name_primer |
| --- | --- | --- | --- |
| LQCBX01TFB | MSR01A01_M13F | LQCBX01TR | MSR01A01_M13R |
| LQCBX02TFB | MSR02A02_M13F | LQCBX02TR | MSR02A02_M13R |
| LQCBX03TFB | MSR03A03_M13F | LQCBX03TR | MSR03A03_M13R |
| LQCBX04TFB | MSR04A04_M13F | LQCBX04TR | MSR04A04_M13R |
| LQCBX05TFB | MSR05A05_M13F | LQCBX05TR | MSR05A05_M13R |
| LQCBX06TFB | MSR06A06_M13F | LQCBX06TR | MSR06A06_M13R |
| LQCBX07TFB | MSR07A07_M13F | LQCBX07TR | MSR07A07_M13R |
| LQCBX08TFB | MSR08A08_M13F | LQCBX08TR | MSR08A08_M13R |
| LQCBX09TFB | MSR09A09_M13F | LQCBX09TR | MSR09A09_M13R |
| LQCBX10TFB | MSR10A10_M13F | LQCBX10TR | MSR10A10_M13R |
| LQCBX11TFB | MSR11A11_M13F | LQCBX11TR | MSR11A11_M13R |
| LQCBX12TFB | MSR12A12_M13F | LQCBX12TR | MSR12A12_M13R |
| LQCBX13TFB | MSR01B01_M13F | LQCBX13TR | MSR01B01_M13R |
| LQCBX14TFB | MSR02B02_M13F | LQCBX14TR | MSR02B02_M13R |
| LQCBX15TFB | MSR03B03_M13F | LQCBX15TR | MSR03B03_M13R |
| LQCBX16TFB | MSR04B04_M13F | LQCBX16TR | MSR04B04_M13R |
| LQCBX17TFB | MSR05B05_M13F | LQCBX17TR | MSR05B05_M13R |
| LQCBX18TFB | MSR06B06_M13F | LQCBX18TR | MSR06B06_M13R |
| LQCBX19TFB | MSR07B07_M13F | LQCBX19TR | MSR07B07_M13R |
| LQCBX20TFB | MSR08B08_M13F | LQCBX20TR | MSR08B08_M13R |
| LQCBX21TFB | MSR09B09_M13F | LQCBX21TR | MSR09B09_M13R |
| LQCBX22TFB | MSR10B10_M13F | LQCBX22TR | MSR10B10_M13R |
| LQCBX23TFB | MSR11B11_M13F | LQCBX23TR | MSR11B11_M13R |
| LQCBX24TFB | MSR12B12_M13F | LQCBX24TR | MSR12B12_M13R |
| LQCBX25TFB | MSR01C01_M13F | LQCBX25TR | MSR01C01_M13R |
| LQCBX26TFB | MSR02C02_M13F | LQCBX26TR | MSR02C02_M13R |
| LQCBX27TFB | MSR03C03_M13F | LQCBX27TR | MSR03C03_M13R |
| LQCBX28TFB | MSR04C04_M13F | LQCBX28TR | MSR04C04_M13R |
| LQCBX29TFB | MSR05C05_M13F | LQCBX29TR | MSR05C05_M13R |
| LQCBX30TFB | MSR06C06_M13F | LQCBX30TR | MSR06C06_M13R |
| LQCBX31TFB | MSR07C07_M13F | LQCBX31TR | MSR07C07_M13R |
| LQCBX32TFB | MSR08C08_M13F | LQCBX32TR | MSR08C08_M13R |
| LQCBX33TFB | MSR09C09_M13F | LQCBX33TR | MSR09C09_M13R |
| LQCBX34TFB | MSR10C10_M13F | LQCBX34TR | MSR10C10_M13R |
| LQCBX35TFB | MSR11C11_M13F | LQCBX35TR | MSR11C11_M13R |
| LQCBX36TFB | MSR12C12_M13F | LQCBX36TR | MSR12C12_M13R |
| LQCBX37TFB | MSR01D01_M13F | LQCBX37TR | MSR01D01_M13R |
| LQCBX38TFB | MSR02D02_M13F | LQCBX38TR | MSR02D02_M13R |
| LQCBX39TFB | MSR03D03_M13F | LQCBX39TR | MSR03D03_M13R |
| LQCBX40TFB | MSR04D04_M13F | LQCBX40TR | MSR04D04_M13R |
| LQCBX41TFB | MSR05D05_M13F | LQCBX41TR | MSR05D05_M13R |
| LQCBX42TFB | MSR06D06_M13F | LQCBX42TR | MSR06D06_M13R |
| LQCBX43TFB | MSR07D07_M13F | LQCBX43TR | MSR07D07_M13R |
| LQCBX44TFB | MSR08D08_M13F | LQCBX44TR | MSR08D08_M13R |
| LQCBX45TFB | MSR09D09_M13F | LQCBX45TR | MSR09D09_M13R |
| LQCBX46TFB | MSR10D10_M13F | LQCBX46TR | MSR10D10_M13R |
| LQCBX47TFB | MSR11D11_M13F | LQCBX47TR | MSR11D11_M13R |
| LQCBX48TFB | MSR12D12_M13F | LQCBX48TR | MSR12D12_M13R |
| LQCBX49TFB | MSR01E01_M13F | LQCBX49TR | MSR01E01_M13R |
| LQCBX50TFB | MSR02E02_M13F | LQCBX50TR | MSR02E02_M13R |
| LQCBX51TFB | MSR03E03_M13F | LQCBX51TR | MSR03E03_M13R |
| LQCBX52TFB | MSR04E04_M13F | LQCBX52TR | MSR04E04_M13R |
| LQCBX53TFB | MSR05E05_M13F | LQCBX53TR | MSR05E05_M13R |
| LQCBX54TFB | MSR06E06_M13F | LQCBX54TR | MSR06E06_M13R |
| LQCBX55TFB | MSR07E07_M13F | LQCBX55TR | MSR07E07_M13R |
| LQCBX56TFB | MSR08E08_M13F | LQCBX56TR | MSR08E08_M13R |
| LQCBX57TFB | MSR09E09_M13F | LQCBX57TR | MSR09E09_M13R |
| LQCBX58TFB | MSR10E10_M13F | LQCBX58TR | MSR10E10_M13R |
| LQCBX59TFB | MSR11E11_M13F | LQCBX59TR | MSR11E11_M13R |
| LQCBX60TFB | MSR12E12_M13F | LQCBX60TR | MSR12E12_M13R |
| LQCBX61TFB | MSR01F01_M13F | LQCBX61TR | MSR01F01_M13R |
| LQCBX62TFB | MSR02F02_M13F | LQCBX62TR | MSR02F02_M13R |
| LQCBX63TFB | MSR03F03_M13F | LQCBX63TR | MSR03F03_M13R |
| LQCBX64TFB | MSR04F04_M13F | LQCBX64TR | MSR04F04_M13R |
| LQCBX65TFB | MSR05F05_M13F | LQCBX65TR | MSR05F05_M13R |
| LQCBX66TFB | MSR06F06_M13F | LQCBX66TR | MSR06F06_M13R |
| LQCBX67TFB | MSR07F07_M13F | LQCBX67TR | MSR07F07_M13R |
| LQCBX68TFB | MSR08F08_M13F | LQCBX68TR | MSR08F08_M13R |
| LQCBX69TFB | MSR09F09_M13F | LQCBX69TR | MSR09F09_M13R |
| LQCBX70TFB | MSR10F10_M13F | LQCBX70TR | MSR10F10_M13R |
| LQCBX71TFB | MSR11F11_M13F | LQCBX71TR | MSR11F11_M13R |
| LQCBX72TFB | MSR12F12_M13F | LQCBX72TR | MSR12F12_M13R |
| LQCBX73TFB | MSR01G01_M13F | LQCBX73TR | MSR01G01_M13R |
| LQCBX74TFB | MSR02G02_M13F | LQCBX74TR | MSR02G02_M13R |
| LQCBX75TFB | MSR03G03_M13F | LQCBX75TR | MSR03G03_M13R |
| LQCBX76TFB | MSR04G04_M13F | LQCBX76TR | MSR04G04_M13R |
| LQCBX77TFB | MSR05G05_M13F | LQCBX77TR | MSR05G05_M13R |
| LQCBX78TFB | MSR06G06_M13F | LQCBX78TR | MSR06G06_M13R |
| LQCBX79TFB | MSR07G07_M13F | LQCBX79TR | MSR07G07_M13R |
| LQCBX80TFB | MSR08G08_M13F | LQCBX80TR | MSR08G08_M13R |
| LQCBX81TFB | MSR09G09_M13F | LQCBX81TR | MSR09G09_M13R |
| LQCBX82TFB | MSR10G10_M13F | LQCBX82TR | MSR10G10_M13R |
| LQCBX83TFB | MSR11G11_M13F | LQCBX83TR | MSR11G11_M13R |
| LQCBX84TFB | MSR12G12_M13F | LQCBX84TR | MSR12G12_M13R |
| LQCBX85TFB | MSR01H01_M13F | LQCBX85TR | MSR01H01_M13R |
| LQCBX86TFB | MSR02H02_M13F | LQCBX86TR | MSR02H02_M13R |
| LQCBX87TFB | MSR03H03_M13F | LQCBX87TR | MSR03H03_M13R |
| LQCBX88TFB | MSR04H04_M13F | LQCBX88TR | MSR04H04_M13R |
| LQCBX89TFB | MSR05H05_M13F | LQCBX89TR | MSR05H05_M13R |
| LQCBX90TFB | MSR06H06_M13F | LQCBX90TR | MSR06H06_M13R |
| LQCBX91TFB | MSR07H07_M13F | LQCBX91TR | MSR07H07_M13R |
| LQCBX92TFB | MSR08H08_M13F | LQCBX92TR | MSR08H08_M13R |
| LQCBX93TFB | MSR09H09_M13F | LQCBX93TR | MSR09H09_M13R |
| LQCBX94TFB | MSR10H10_M13F | LQCBX94TR | MSR10H10_M13R |
| LQCBX95TFB | MSR11H11_M13F | LQCBX95TR | MSR11H11_M13R |
| LQCBX96TFB | MSR12H12_M13F | LQCBX96TR | MSR12H12_M13R |
