# Supplementary material for: Construction and sequence sampling of deep-coverage, large-insert BAC libraries for three model lepidopteran species
Source: BMC Genomics. 2009 Jun 26;10:283. doi: 10.1186/1471-2164-10-283 (PMC2718931; doi:10.1186/1471-2164-10-283)
Supplement: Additional file 4 — Detailed repeat element contents in the BESs of three lepidopteran models. Table S4 summarizes the types of repeat elements found in the BESs of H. erato, H. virescens, and M. sexta. [file 1471-2164-10-283-S4.doc]

Table S4. Detailed repeat element contents in the BESs of three lepidopteran models. Hv, *Heliothis virescens*; He, *Heliconius erato*; Ms, *Manduca sexta*.

| Repeat class/family | Repeat type | No. of repeats | | |
| --- | --- | --- | --- | --- |
|  |  | Hv | He | Ms |
| Low_complexity | AT_rich | 76 | 207 | 102 |
|  | A_rich | 1 |  | 1 |
|  | GC_rich | 2 |  |  |
|  | GA-rich |  | 1 |  |
|  | T-rich |  | 1 |  |
| Simple_repeat | (CAGA)n | 2 | 1 | 1 |
|  | (A)n | 4 | 1 |  |
|  | (TTTA)n | 1 | 1 |  |
|  | (CAA)n | 1 |  |  |
|  | (CAAA)n | 4 |  |  |
|  | (CGAAG)n | 1 |  |  |
|  | (G)n | 1 |  |  |
|  | (GTATG)n | 1 |  |  |
|  | (TA)n | 2 |  |  |
|  | (TTA)n | 1 |  |  |
|  | (TTTTTA)n | 1 |  |  |
|  | (TTTG)n | 1 |  | 1 |
|  | (TCTG)n | 2 |  | 1 |
|  | (CTATT)n |  | 1 |  |
|  | (TAA)n |  | 1 |  |
|  | (TAAA)n |  | 2 |  |
|  | (TATG)n |  | 1 |  |
|  | (CA)n |  | 2 |  |
|  | (CATA)n |  | 1 |  |
|  | (CCA)n |  | 1 |  |
|  | (TTAAA)n |  | 1 |  |
|  | (TTTTA)n |  | 1 |  |
|  | (T)n |  | 2 | 2 |
|  | (TTATA)n |  | 1 | 1 |
|  | (AAATG)n |  |  | 1 |
|  | (CGGG)n |  |  | 1 |
|  | (TC)n |  |  | 1 |
|  | (TG)n |  |  | 1 |
|  | (TTCA)n |  |  | 1 |
| SINE/tRNA | BM1 |  |  | 4 |
| SINE? | BM1B |  |  | 3 |
| LINE/R1 | TRAS3_BM | 1 |  |  |
| LINE/Jockey | LINE1_BM |  | 1 |  |
| LTR/Gypsy | DM297_I | 1 |  |  |
| LTR/Gypsy | Gypsy8-I_AG | 1 |  |  |
| LTR/Gypsy | Gypsy24-I_AG |  |  | 2 |
| LTR/Gypsy | Gypsy52-I_AG |  | 1 |  |
| LTR/Copia | DM1731_I |  |  | 1 |
| DNA/Mariner | Mariner_HC |  | 2 | 1 |
| RC/Helitron | Helitron2_AG |  | 1 |  |
| rRNA | LSU-rRNA_Dme | 4 |  |  |
| rRNA | SSU-rRNA_Dme |  |  | 1 |
| Total | | 108 | 231 | 126 |
